# Spectral Computed Tomography Diagnosis of Inflammatory Bowel Disease with Neodymium-Hyaluronic Acid Nanoparticles

**DOI:** 10.34133/bmr.0301

**Published:** 2026-03-25

**Authors:** Xin Zhu, Wenqian Ru, Lin Luo, Liu Zhao, Didi Gu, Xi Deng, Yihan Wang, Pengyi Huang, Qiuyu Meng, Chunmei Yang, Lu Yang

**Affiliations:** ^1^ Department of Radiology, The Affiliated Hospital, Southwest Medical University, Luzhou Precision Imaging and Intelligent Analysis Key Laboratory of Luzhou, Luzhou, Sichuan, 646000, China.; ^2^ Key Laboratory of Pollution Exposure and Health Intervention of Zhejiang Province, Interdisciplinary Research Academy (IRA), Zhejiang Shuren University, Hangzhou 310015, China.; ^3^ Department of Endocrinology and Metabolism, The Affiliated Hospital of Southwest Medical University, Metabolic Vascular Disease Key Laboratory of Sichuan Province, and Sichuan-Chongqing Joint Key Laboratory of Metabolic Vascular Diseases, Luzhou, Sichuan, 646000, China.

## Abstract

Early detection and nonintrusive assessment of inflammatory bowel disease (IBD) remain an unmet clinical challenge. Spectral computed tomography (CT) presents a potential modality for gastrointestinal (GI) imaging; however, clinical CT contrast agents are unable to achieve targeted detection of IBD in spectral CT imaging. In this study, we developed neodymium-hyaluronic acid nanoparticles (Nd-HA NPs) as novel contrast agents for spectral CT imaging of IBD. Nd-HA NPs were synthesized by conjugating HA units with lanthanide complex neodymium-diethylenetriamine-pentaacetic acid (Nd-DTPA). The physical properties, biotoxicity, and CT imaging ability of Nd-HA NPs were systematically evaluated in vitro. Subsequently, the applicability of Nd-HA NPs for GI tract imaging was assessed in both healthy and colitis mouse models. Nd-HA NPs exhibited excellent stability, biocompatibility, and potent x-ray attenuation property in vitro as novel spectral CT contrast agents. Attributed to HA’s high affinity for cluster of differentiation 44 receptor, which is abundantly expressed at inflammatory sites, Nd-HA NPs successfully achieved targeted spectral CT imaging of IBD, and showed greater accumulation in the lesions of colitis mice compared with the clinical contrast agent iohexol. More importantly, after oral administration of Nd-HA NPs, the CT values of GI tract in healthy mice, 2.5% DSS-induced mice (moderate colitis), and 5% DSS-induced mice (severe colitis) were 90.19, 140.99, and 264.07 HU, respectively, with statistically significant difference (*P* < 0.001). These results indicated that Nd-HA NPs had the potential to realize severity assessment of IBD in spectral CT imaging, which was further confirmed by inductively coupled plasma optical emission spectrometry analysis and histopathological evaluation. The study suggested that Nd-HA NPs could serve as effective spectral CT contrast agents, enabling noninvasive early detection and severity assessment of IBD.

## Introduction

Inflammatory bowel disease (IBD) is a chronic gastrointestinal (GI) disorder, primarily comprising Crohn’s disease (CD) and ulcerative colitis (UC) [1,2]. The persistent clinical symptoms of IBD include frailty, recurrence, high heterogeneity, immune dysregulation, and a tendency for disease progression with no available curative therapy [3]. The pathogenesis of IBD remains unknown but is thought to involve a combination of environmental, immune, and genetic factors [4,5]. Beyond causing considerable clinical morbidity, IBD elevates colorectal carcinogenesis risk through sustained inflammatory responses [6]. Moreover, with the progress of IBD, 25% to 40% of IBD patients develop extra-intestinal symptoms such as arthritis, skin diseases, scleritis, and primary sclerosing cholangitis [7]. Therefore, early diagnosis and severity assessment of IBD are crucial for reducing the burden on patients [8].

Currently, the standard for diagnosing IBD is histopathological examination; however, the method is invasive and unsuitable for follow-up studies of patients [9]. Several noninvasive methods lack the specificity and accuracy in IBD assessment. For example, overlapping GI tissues and gas interference make it difficult for ultrasound to depict the intestinal structures [10], and magnetic resonance imaging (MRI) is neither cost-effective nor friendly to GI monitor [11]. Conversely, computed tomography (CT) provides significant advantages including noninvasiveness, low cost, strong tissue penetration, and rapid image acquisition, making it a foremost modality for GI imaging [12,13]. Functioning as an advanced CT modality, spectral CT distinguishes between normal and contrast-enhanced tissues by leveraging differential x-ray attenuation coefficients [14]. It further provides both qualitative and quantitative analysis of tissue composition via material-decomposition images [15]. In addition, by acquiring a series of virtual monochromatic images, spectral CT facilitates the identification of optimal energy levels to improve image contrast and obtain more precise characterization of lesions [16]. Consequently, spectral CT has been employed in the monitor of various disease, such as distinguishing small hepatic hemangiomas from small hepatocellular carcinomas [17], improving the diagnostic sensitivity of insulinomas [18], and evaluating the severity in ileocolonic CD [19]. Despite the numerous advantages of spectral CT, the imaging performance of current contrast agents is unsatisfactory. Consequently, it is crucial to develop a next-generation contrast agent to promote the specific diagnosis of IBD in spectral CT imaging.

The unique 4f electronic configuration of lanthanide elements confers multifunctional optical, electronic, and magnetic properties, making them ideal candidate materials for the development of advanced contrast agents for multimodal imaging [20,21]. Lanthanide metals, such as Ho [22], Gd [23], Nd [24,25], and Yb [26], have been applied in MRI, optical imaging, and CT imaging. It is reported that elements with higher atomic numbers show superior x-ray absorption, and materials with higher K-edge values are beneficial for the improvement of contrast resolution in spectral CT imaging. Accordingly, neodymium (Nd), a lanthanide element with an atomic number of 60 (*Z* = 60) and a K-edge value of 43.5 keV, demonstrates great potential for spectral CT imaging, which is better than clinical iodinated contrast agents (I, *Z* = 53, K-edge value = 33.2 keV). Compared with conventional contrast agents that require larger doses and may cause nephrotoxicity [27], Nd-based contrast agents with stronger x-ray absorption and superior x-ray attenuation properties represent a promising choice of GI examination in spectral CT imaging.

However, traditional CT contrast agents have no specificity for inflammation and cannot accumulate at the inflammatory sites, leading to the difficulty of accurate diagnosis and grading of IBD. Cluster of differentiation 44 (CD44) is widely expressed in the inflammatory infiltration of colitis in mice and humans [28]; therefore, targeting CD44 receptors is considered an effective means of IBD diagnosis and treatment [29,30]. Hyaluronic acid (HA) has strong affinity toward the CD44 receptor, endowing HA-loaded drugs favorable inflammation targeting ability, improving drug efficacy, and reducing systemic side effects greatly [31,32]. In addition, as an inherent linear polysaccharide in the human body, HA has the advantages of hydrophilicity, biodegradability, and nonimmunogenicity; thus, it is widely utilized as an intelligent carrier for anti-inflammation drugs. Moreover, several HA-loaded contrast agents have been utilized in CT and MRI of articular cartilage [33], liver, and neoplasm [34,35]. At present, artificial contrast agents are mainly divided into nanoparticles and small molecules. The former is an ideal choice for GI imaging, because they can carry higher amounts of contrast agents, possess passive targeting of inflammation, and have long half-time in the bloodstream [36,37]. Therefore, the development of HA-loaded nanocontrast agents is expected to allow for targeted imaging-based monitoring of IBD.

**Fig. 1.**
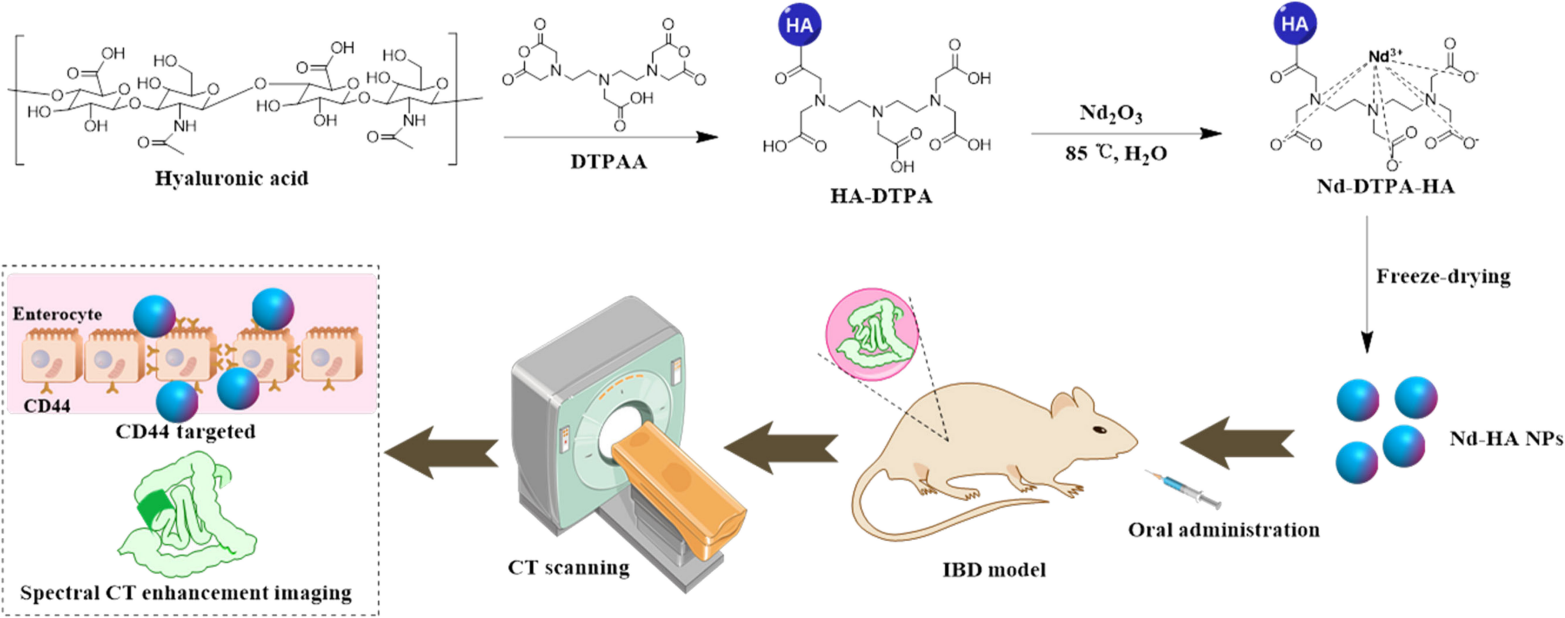
Schematic illustration of the fabrication of Nd-HA NPs as targeted spectral CT contrast agents for IBD imaging in vivo.

In this study, based on the advantages of the Nd element and HA component, we developed neodymium-HA nanoparticles (Nd-HA NPs) as novel spectral CT contrast agents for the early diagnosis of IBD. Nd-HA NPs were obtained in high yield using a facile and green one-pot synthesis strategy (Fig. 1). We first evaluated the chemical characteristics, toxicity, and in vitro spectral CT imaging ability of Nd-HA NPs. Given the outstanding imaging potential and good biocompatibility of Nd-HA NPs, they were further applied to the in vivo spectral CT imaging of BALB/c mice, and the clinical CT contrast agent iohexol was used as a comparison. Spectral CT imaging, pathological examination, and inductively coupled plasma optical emission spectrometry (ICP-OES) analysis were performed on healthy mice and colitis mice with different severity levels to find out whether Nd-HA NPs could achieve the specific accumulation, early diagnosis, and severity assessment of IBD.

## Materials and Methods

### Materials and reagents

All the chemicals and reagents were analytical grade and used without further purification. Sodium hyaluronate (HA, MW ≈ 1,500 to 1,800 kDa) was obtained from Sigma-Aldrich, diethylenetriaminepentaacetic dianhydride (DTPAA) was purchased from TCI. Neodymium (III) oxide (Nd_2_O_3_) and dimethyl sulfoxide (DMSO) were obtained from Acros. PBS buffer and penicillin–streptomycin solution were purchased from Beyotime Biotechnology. Dulbecco’s minimum essential medium (DMEM) and Roswell Park Memorial Institute-1640 (RPMI-1640) medium were purchased from GIBCO. Fetal bovine serum (FBS) was obtained from Lonsera.

### Synthesis of Nd-HA NPs

Nd-HA NPs were prepared using a modified protocol according to previous procedures [33]. Firstly, 400 mg of sodium hyaluronate was dissolved in 40 ml of deionized (DI) water and stirred vigorously for 1 h. After that, 180 mg of DTPAA in 1 ml of DMSO was added rapidly to the solution and stirred for another 6 h to obtain HA-DTPA composite. Next, 84.2 mg of Nd_2_O_3_ (140 mg) in 2 ml of H_2_O was added dropwise and stirred vigorously at 85 °C until the mixture turned transparent. The resulting solution was further dialyzed with DI water for 24 h. Finally, the purified Nd-HA NPs solution was subjected to a standard freeze-drying procedure and stored at 4 °C for further study.

### Characterization

Transmission electron microscopy (TEM) and energy dispersive spectroscopy (EDS) analysis of the morphological, size, and element mappings of Nd-HA NPs were performed on Tecnai G2F30 (PEI, USA). Fourier transform infrared spectroscopy (FT-IR) spectra were confirmed by a Nicole 6700 FT-IR spectrometer (Thermo Fisher, USA). ICP-OES (Agilent, USA) was utilized to evaluate the content of Nd element in Nd-HA NPs. X-ray photoelectron spectroscopy (XPS) patterns of Nd-HA NPs were obtained using a Thermo escalab 250Xi instrument with Al Kα (*hν* =1,486.6 eV) x-ray radiation. ^1^H-nuclear magnetic resonance (NMR) spectra of HA, HA-DTPA, and Nd-HA NPs were measured on a Bruker Biospin AV400 (400 MHz) instrument.

### Structural stability of Nd-HA NPs

To test the colloidal stability of Nd-HA NPs, we dissolved them in different media, e.g., normal saline, RPMI-1640, DMEM, PBS, and FBS at 37 °C for 1 and 7 days, respectively, then detected if there were precipitates or aggregates formed. After that, the leakage of Nd^3+^ in Nd-HA NPs was investigated using the xylenol orange (XO) indicator (0.2%). Additionally, to evaluate whether Nd-HA NPs would survive the resistance of the GI tract, they were exposed to different pH buffer solutions (pH = 2.2 and pH = 8.0) for 2 days to mimic the harsh environment of stomach and small intestine, then ICP-OES was used to analyze the leakage of Nd^3+^.

### Cytotoxicity assessment

CCK-8 assay was conducted to evaluate the cytotoxicity of Nd-HA NPs. 4T1 and MCF-10A cells were cultured in RPMI-1640 complete medium (containing 10% fetal FBS) and DMEM complete medium (containing 10% FBS), respectively. The cells were plated in 96-well plates at densities of 8 × 10^3^ per well and cultured for 24 h (37 °C/5% CO_2_). Next, different concentrations of Nd-HA NPs solutions (0, 12.5, 25, 50, 100, 200, and 400 μg/ml) were added to the plates and incubated for another 24 h. After removing the medium, 10 μl of CCK-8 was added to each well. Absorbance was measured after 1 h incubation using a microplate reader.

### In vivo toxicity assessment

All the animal experiments were carried out following the guidelines and protocols of the Animal Care and Use Committee of Southwest Medical University (Project number: 20231011-001) and conducted according to the *Guide for the Care and Use of Laboratory Animals, 8th edition* published by NIH. Six- to 8-week-old female BALB/c mice were purchased from Chongqing Tengxin Huafu Experimental Animal Sales Co. Ltd (Chongqing, China) and randomly divided into 3 groups (*n* = 4). After 1 week of acclimatization, the experimental groups were given 0.2 ml of Nd-HA NPs solution (0.1 M), and then sacrificed on the 1st and 10th day, respectively, for biochemical analysis and hematoxylin and eosin (H&E) staining. Mice given 0.2 ml of normal saline were used as control. Blood samples obtained from the mice were used for the determination of several important biomarkers: alanine aminotransferase (ALT), aspartate aminotransferase (AST), alkaline phosphatase (ALP), serum albumin, creatinine (CREA-S), uric acid (UA), urea (UREA), and total protein. Main organs including the heart, liver, spleen, lungs, kidneys, stomach, small intestine, and large intestine were collected and fixed in a 4% paraformaldehyde solution. H&E staining was then used to assess the histopathological changes of the mice.

### Flow cytometric analysis of CD44

RAW264.7 cells were seeded in 6-well plates and stimulated with 1 μg/ml LPS (lipopolysaccharide) for 24 h. Subsequently, the cells were stained with a fluorescently labeled anti-CD44 antibody and analyzed by flow cytometry.

### Cellular targeting experiment

RAW264.7 cells were seeded in culture medium and stimulated with 1 μg/ml LPS for 24 h to establish an inflammatory model. The groups were set as follows: control, Nd-DTPA, Nd-HA NPs (unblock group), and Nd-HA NPs + HA (block group). For the block group, cells were exposed to HA (5 mg/ml) for 4 h, then incubated with Nd-HA NPs at concentrations of 0, 250, 500, and 1,000 μg/ml for an additional 4 h. The other groups were treated with corresponding compounds for 4 h accordingly. All other groups were treated with their respective compounds for 4 h. After treatment, cells were harvested, digested, and prepared for CT imaging to evaluate nanoparticle uptake and receptor-specific targeting.

### In vitro spectral CT imaging

Nd-HA NPs and iohexol (0, 0.0125, 0.025, 0.05, and 0.1 M Nd/I) were imaged by spectral CT (Philips IQON; 150 × 150 mm field of view [FOV], 0.4 mm slices, 100 mA, 120 keV) to evaluate the in vitro spectral CT imaging ability. Virtual monochromatic images (40 to 180 keV, 20-keV steps) were generated using Philips Intellispace Portal Workstation.

### IBD modeling

According to the previous report, the IBD models were constructed by adding different concentrations of dextran sodium sulfate (DSS) in the drinking water of 6- to 8-week-old female BALB/c mice [38]. The mice were randomly divided into 3 groups. Severe group: fed with 5% (w/v) DSS solution for 7 days and tap water for 1 day; moderate group: fed with 2.5% (w/v) DSS solution for 7 days and tap water for 1 day; control group: fed with tap water for 8 days. The clinical symptoms of IBD in each group were observed at a fixed time every day, including weight changes and defecation. Clinical IBD score was used to measure disease severity: 1 point for weight loss of less than 3%, 2 points for weight loss of 4% to 10%, 3 points for weight loss of 11% to 20%, and 4 points for weight loss of more than 20%. In terms of bowel movements, 0 points for diarrhea, 1 point for loose stools, and 2 points for visible blood in stools. Therefore, the overall clinical IBD score ranged from 0 to 6 points.

### In vivo spectral CT

Six- to 8-week-old female BABL/c mice were kept in 50% ± 5% humidity in a 20 to 25 °C environment, accompanied by a light–dark cycle of 12 h and with free access to water and food. Colitis mice with varying severity degrees were induced as above. To evaluate the imaging performance and inflammation targeting ability of Nd-HA NPs, healthy mice and 5% DSS-induced mice (severe colitis) were orally administrated with 0.1 M Nd-HA NPs, iohexol, and Nd-DTPA solution, respectively (*n* = 3). In addition, the moderate group was given 0.1 M Nd-HA NPs to confirm whether they had the ability to assess the severity of IBD (*n* = 3). After isoflurane anesthesia (1.5% to 2.5%, 0.8 ml/min O_2_), mice were then subjected to spectral CT scans (Philips IQON; 150 × 150 mm FOV, 0.4 mm slices, 120 keV, 100 mA) pre- and post-contrast administration.

### Pathological analysis

After spectral CT imaging, colons (proximal rectum to ileocecal junction) were harvested, PBS-perfused, and longitudinally measured. Histopathological alterations were assessed via H&E and Masson’s trichrome staining. The specimens were observed under a microscope and the results were evaluated according to the IBD pathology scoring criteria [39]: 0 points for normal specimens with no visible gross lesions, intact mucosa, and an absence of inflammatory cells; 1 point for specimens with a limited number of focal and mononuclear inflammatory cells within the lamina propria, along with crypt hyperplasia; 2 points for specimens with mild inflammation, characterized by a small, multifocal mononuclear infiltrate with few neutrophils and epithelial cell hyperplasia; 3 points for specimens with moderate inflammations, marked by extensive multifocal monocyte and neutrophil infiltration, crypt abscess formation, and increased epithelial hyperplasia; 4 points for specimens with severe inflammation, characterized by severe diffuse infiltration of mononuclear cells, a significant amount of neutrophils, transmural inflammation, reduced mucin levels, and the presence of crypt abscesses. The collected colon tissues were also analyzed using ICP-OES to measure the concentration of Nd element in the lesions.

### Pharmacokinetics and biodistribution analysis

After oral administration of 0.2 ml of 0.1 M Nd-HA NPs to healthy mice, tissue samples including heart, liver, spleen, lung, kidney, stomach, small intestine, and large intestine were collected at 0, 0.5, and 24 h for ICP-OES detection.

### Statistical analysis

The data obtained were analyzed using SPSS 26 (IBM, USA), and error bars were expressed as standard errors. All data were initially subjected to normality and variance analyses, followed by evaluation using the one-way ANOVA statistical analysis to evaluate the significance of the experimental data. *P < 0.05, **P < 0.01, ***P < 0.001.

## Results

### Synthesis and characterization of Nd-HA NPs

**Fig. 2.**
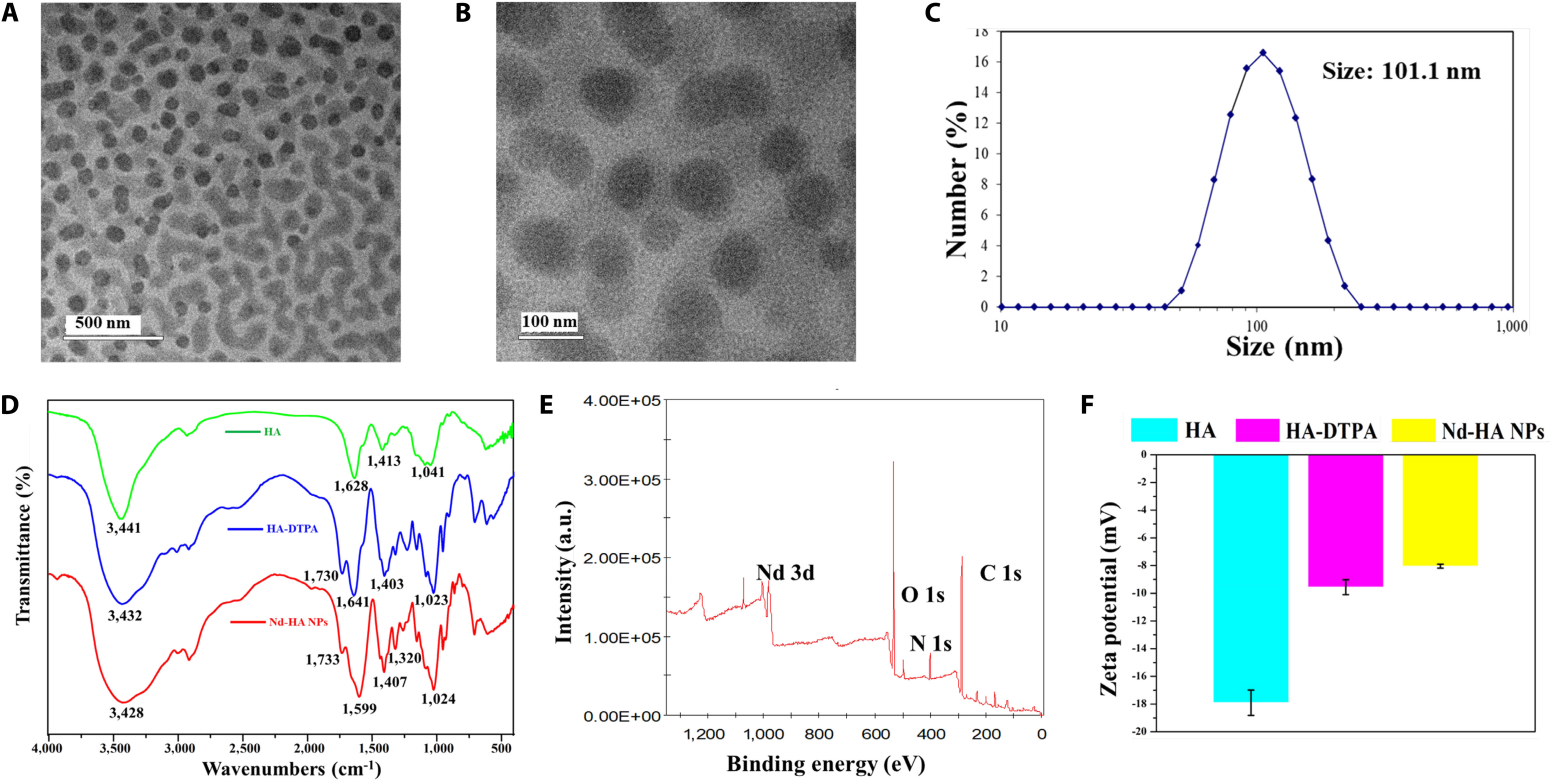
Characterization of Nd-HA NPs. (A and B) TEM images of Nd-HA NPs. (C) Hydrodynamic size. (D) FT-IR spectra of HA, HA-DTPA, and Nd-HA NPs. (E) XPS spectrum of Nd-HA NPs. (F) The zeta potential of HA, HA-DTPA, and Nd-HA NPs.

Nd-HA NPs were obtained in high yield (95%) through a facile method. As shown in Fig. 1, HA firstly reacted with DTPAA to get HA-DTPA via a ring-opening reaction. DTPA acts as a high-affinity chelating ligand for Nd^3+^ ions, effectively preventing metal ion leakage. Then, Nd_2_O_3_ solution was added dropwise into the mixture to produce Nd-DTPA-HA composite. After that, the solution was dialyzed and freeze-dried to yield Nd-HA NPs. The content of Nd element in Nd-HA NPs was determined to be 9.6% by ICP-OES. The morphology and size of Nd-HA NPs were characterized by TEM (Fig. 2A to C), which indicated that they were spherical nanoparticles with a uniform distribution and an average diameter of 100.7 ± 0.6 nm. EDS spectrum further demonstrated the presence and distribution of Nd, C, N, and O elements in Nd-HA NPs (Fig. S1). Additionally, HA, HA-DTPA, and Nd-HA NPs were detected via FT-IR. As shown in Fig. 2D, for pure HA and HA-DTPA, the peaks at 1,628 and 1,641 cm^−1^ were ascribed to the stretching vibration of C=O (-COOH), while in Nd-HA NPs, the main characteristic absorption peak of C=O stretching vibration shifted to 1,599 cm^−1^, validating the coordination of COO- with Nd^3+^ and the formation of Nd chelate. XPS analysis confirmed the binding energies of Nd, O, N, and C elements in Nd-HA NPs (Fig. 2E), and according to the high-resolution XPS spectra of Nd-HA NPs (Fig. S2), characteristic peaks at 981.5, 531.84, 399.52, and 285.14 were assigned to the binding energies of Nd 3d, O 1s, N 1s, and C 1s, respectively. Finally, ^1^H-NMR analyses of HA, HA-DTPA, and Nd-HA NPs were used to confirm the coordination of COO^−^ with Nd^3+^. As shown in Fig. S3, the protons (-CH_2_-) of Nd-HA NPs between carbonyl and tertiary amine shifted to the lower field, owing to the deshielding effect produced by the chelate reaction between Nd (III) and DTPAA. The surface electronegativity of Nd-HA NPs increased because of the introduction of DTPAA and Nd^3+^ that have positive zeta potential (Fig. 2F). These results confirmed the successful synthesis of Nd-HA NPs.

After that, Nd-HA NPs were further dissolved in different media, e.g., normal saline, RPMI-1640, DMEM, PBS, and FBS, and the solutions were kept at 37 °C for 1 and 7 days to test the colloidal stability of Nd-HA NPs. There were no precipitates or aggregates formed, suggesting the excellent structural stability of Nd-HA NPs (Fig. S4A and B). The yellow XO and free Nd^3+^ can form a red complex when the concentration of Nd^3+^ was as low as 0.02 mM. However, almost no red complex was found in the mixture of XO and 100 mM Nd-HA NPs, pointing out that there was no obvious leakage of Nd^3+^ (Fig. S4c). Moreover, Nd-HA NPs were exposed to different pH buffer solutions (pH = 2.2 and pH = 8.0) for 2 days to evaluate whether they could stand the harsh environment of the GI tract, and negligible leakage of Nd^3+^ was detected by ICP-OES (Table S1), suggesting that Nd-HA NPs had outstanding structural stability.

### Toxicity evaluation of Nd-HA NPs

**Fig. 3.**
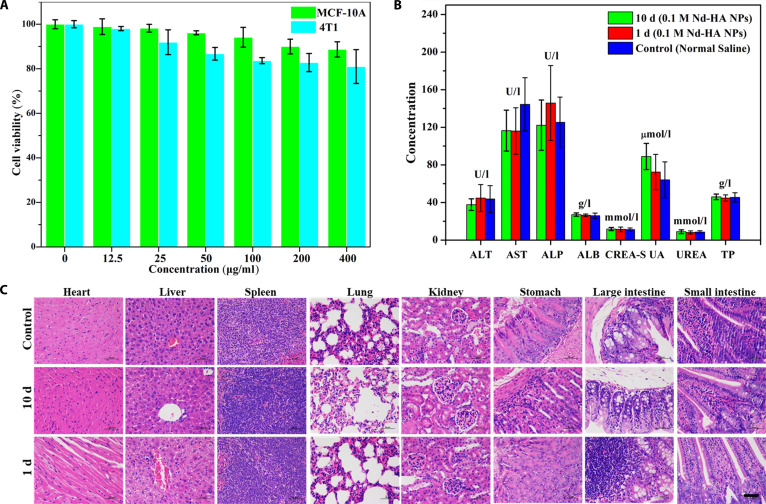
In vitro and in vivo toxicity evaluation of Nd-HA NPs. (A) Cell viability of MCF-10A and 4T1 cells after incubation with Nd-HA NPs (0, 12.5, 25, 50, 100, 200, and 400 μg/ml) for 24 h. (B) Typical biomarkers of healthy mice and Nd-HA NPs-treated mice (250 mg of Nd/kg body weight). (C) H&E staining of heart, liver, spleen, lung, kidney, large intestine, small intestine, and stomach of healthy mice and Nd-HA NPs-treated mice (250 mg of Nd/kg body weight), tested on the 1st and 10th day after Nd-HA NPs or saline administration. Scale bar: 200 μm.

CCK-8 assay was performed to test the cytotoxicity of Nd-HA NPs. Different concentrations (0, 12.5, 25, 50, 100, 200, and 400 μg/ml) of Nd-HA NPs solution were added to MCF-10A and 4T1 cells, respectively, and the cell viability was measured 24 h post-administration (Fig. 3A). The result indicated that both 4T1 and MCF-10A cells were not adversely affected by Nd-HA NPs since the cell viability was above 80% at all conditions, indicating that Nd-HA NPs had extremely low cytotoxicity.

Systemic toxicity was assessed via biochemical assays and H&E histopathology. Female BALB/c mice (6 to 8 weeks) received oral gavage of 0.1 M Nd-HA NPs: acute (1 day) and chronic (10 days) exposure groups. Major organs and blood were harvested for comparative histopathological analysis against saline-treated controls. As expected, there were no notable differences between normal mice and Nd-HA NPs treated mice in regard to the liver function markers of ALT, AST, and ALP, and the renal function markers of CREA-S, UA, and UREA (Fig. 3B), proving that Nd-HA NPs did not cause any damage to the liver and kidney when administrated. H&E staining of vital organs, such as heart, liver, spleen, lung, kidney, large intestine, small intestine, and stomach showed that no pathological signs of hemorrhage, inflammation, and necrosis were observed in the control and experimental groups (Fig. 3C). Meanwhile, no obvious behavioral changes appeared after Nd-HA NPs treatment. These results indicated that Nd-HA NPs had good biocompatibility, which was beneficial for in vivo spectral CT imaging of IBD.

### In vitro targeting assessment

RAW264.7 cells were stimulated with 1 μg/ml LPS for 24 h, and flow cytometry results showed a marked increase in CD44 expression in RAW264.7 cells, indicating the successful establishment of an inflammatory mode (Fig. S5). A concentration-dependent increase in CT values was observed following the addition of Nd-HA NPs at concentrations of 0, 250, 500, and 1,000 μg/ml. To determine whether this enhancement was mediated by CD44 targeting, a competitive binding assay was conducted using free HA as a blocking agent. After pre-incubation with HA, the concentration-dependent increase in CT values was abolished (Fig. S6). Furthermore, neither the Nd-DTPA group nor the noninduced inflammatory group exhibited a substantial elevation in CT signals. These results confirm that the HA-functionalized Nd-HA NPs specifically target inflammatory cells through CD44 receptor-mediated binding.

### In vitro spectral CT imaging of Nd-HA NPs

**Fig. 4.**
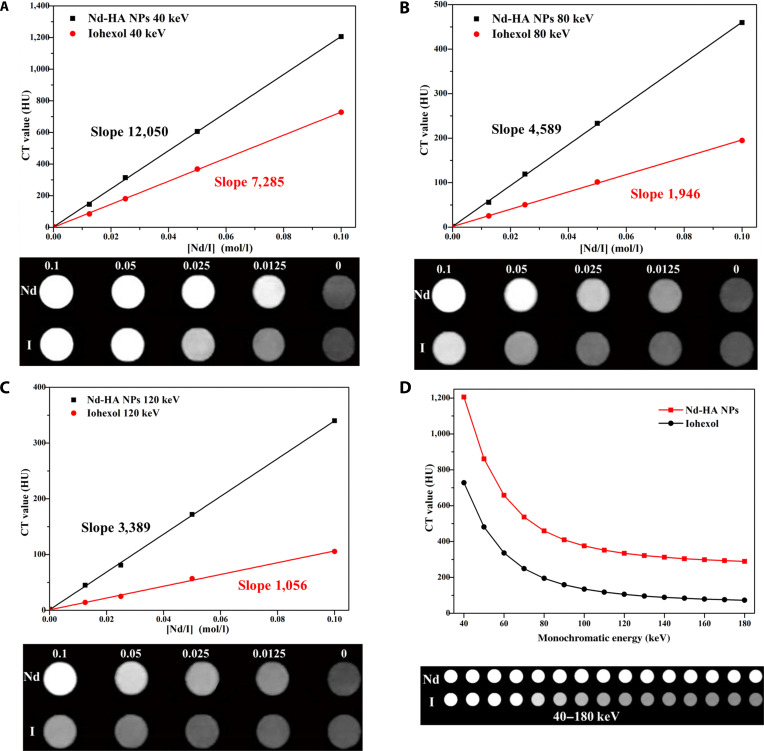
Spectral CT images and HU curves of different concentrations (0, 0.0125, 0.025, 0.05, 0.1 M Nd or I) of Nd-HA NPs vs. iohexol at (A) 40 keV, (B) 80 keV, (C) 120 keV, and (D) 0.1 M Nd-HA NPs and 0.1 M iohexol at different monochromatic energies.

Next, spectral CT imaging of different concentrations (0, 0.0125, 0.025, 0.05, and 0.1 M) of Nd-HA NPs solutions was performed in vitro with isoconcentration iohexol as a control. At monochromatic energies of 40, 80, and 120 keV, the CT values increased linearly with the rising concentration of the contrast agents (Fig. 4A to C). At 40 keV, bright images were observed in the Nd-HA NPs group even if the concentration was as low as 0.0125 M, while iohexol showed comparable imaging effects at a much higher concentration of 0.05 M. Moreover, it was worth noting that under the same monochromatic energy, the CT value and image brightness of Nd-HA NPs were all higher than those of equivalent iohexol. These results indicated that Nd-HA NPs displayed higher x-ray absorption and generated brighter CT images than iohexol, which may reduce the usage of contrast agents when administrated. Progressive signal attenuation was observed at elevated monochromatic energies (40 to 180 keV) in all experimental groups; however, the brightness of iohexol was too low to meet the imaging requirement of spectral CT when the monochromatic energies exceeded 80 keV. In contrast, Nd-HA NPs exhibited higher CT values and produced much brighter spectral CT images at high monochromatic energy levels, suggesting that Nd-HA NPs possessed superior x-ray attenuation coefficient compared to iohexol (Fig. 4D and Table S2). These results consistently verified that Nd-HA NPs showed outstanding spectral CT imaging performance in vitro.

### In vivo CT imaging of healthy mice

**Fig. 5.**
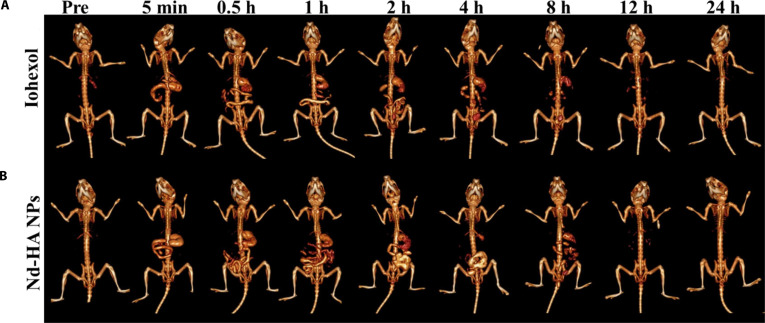
In vivo CT imaging of GI tract in healthy mice after oral administration of (A) 0.1 M iohexol and (B) 0.1 M Nd-HA NPs.

Given the excellent biocompatibility and in vitro spectral CT imaging performance, Nd-HA NPs were applied to GI tract imaging of healthy mice with equivalent iohexol as a comparison (*n* = 3). As shown in Fig. 5 and Fig. S7, 3D reconstructions were generated at serial time points (pre-contrast to 24 h post-administration: 5 min, 0.5 h, 1 h, 2 h, 4 h, 8 h, 12 h, and 24 h). After Nd-HA NPs administration, bright signals were observed in the stomach and duodenum at 5 min, and a notable CT enhancement appeared in the jejunum and ileum at 30 min. Later on, the signals were faded gradually; Nd-HA NPs were nearly emptied from the upper GI tract at 12 h and completely emptied at 24 h. The results suggested that Nd-HA NPs were able to depict the complete GI structures within 0.5 h and be fully metabolized within 24 h. However, several details of the GI tract were missing in iohexol-treated mice, demonstrating that Nd-HA NPs had superior CT imaging performance than iohexol. Additionally, spectral CT imaging using barium sulfate was performed in normal mice. As a suspension, barium sulfate is prone to sedimentation in the GI lumen, resulting in discontinuous and nonuniform distribution along the tract (Fig. S8).

**Fig. 6.**
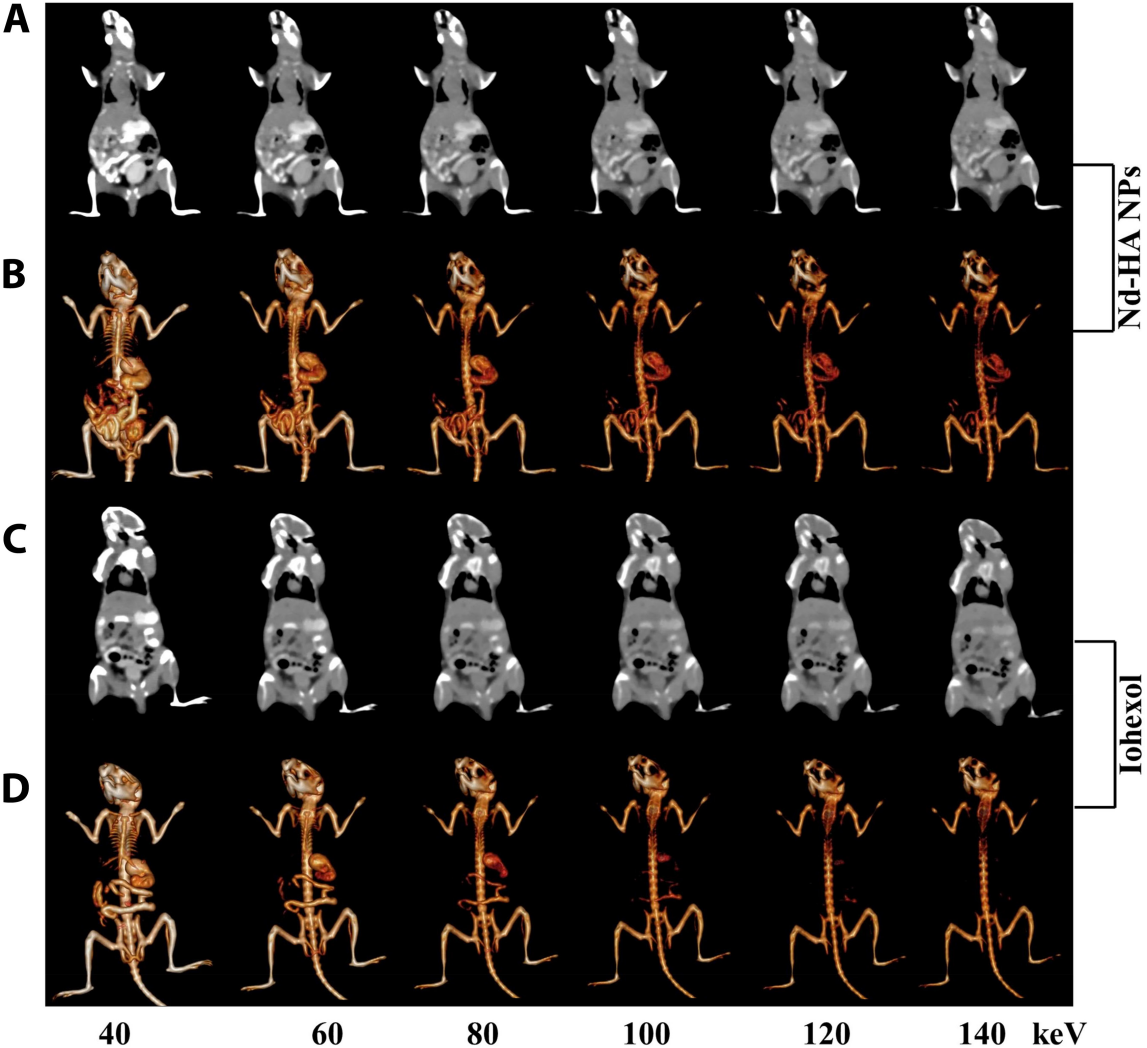
In vivo spectral CT imaging of GI tract in healthy mice after oral administration of 0.1 M Nd-HA NPs and iohexol for 0.5 h, respectively. (A) Gray-scale coronal CT images and (B) 3D reconstruction images of Nd-HA NPs-treated mice. (C) Gray-scale coronal CT images and (D) 3D reconstruction images of iohexol-treated mice.

In order to obtain more GI information, the gray-scale coronal images and 3D reconstruction images at different monochromatic energies were acquired by post-processing the scanned images (Fig. 6 and Fig. S9). The brightest images were obtained at 40 keV for both Nd-HA NPs and iohexol, but only the former had the ability to fully describe the GI structures of healthy mice. On the other hand, CT signals decreased in both groups with the increasing monochromatic energies, but the decline was more severe in iohexol-treated mice, and the profile of the GI tract was hardly visible at monochromatic energies above 100 keV. In contrast, CT attenuation of Nd-HA NPs was much milder; not only could the GI tract be seen at all monochromatic energies, but the images at 40, 60, and 80 keV also had favorable examination significance. These results indicated that Nd-HA NP had superior x-ray attenuation compared with iohexol, displaying better performance in both conventional and spectral CT imaging of the GI tract in healthy mice.

### IBD modeling

**Fig. 7.**
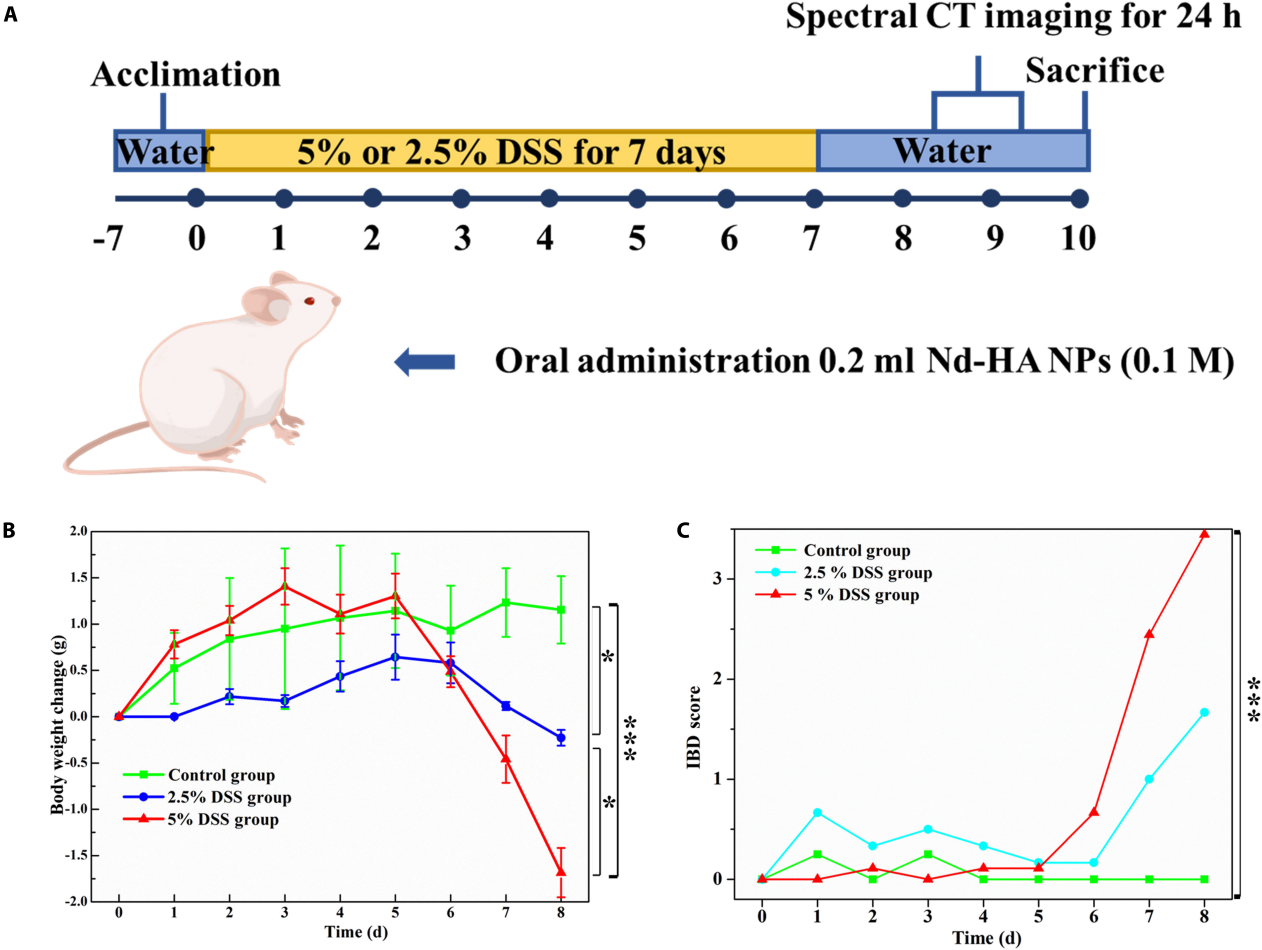
(A) Schematic illustration of colitis mice modeling. (B) Body weight fluctuations of healthy mice, 2.5% and 5% DSS-treated mice within 8 days. Data were expressed as mean ± SEM. (C) Disease activity index (DAI) expressed as IBD score of healthy mice, moderate colitis mice (2.5% DSS), and severe colitis mice (5% DSS). *P < 0.05, ***P < 0.001.

Colitis mouse models were constructed by feeding the mice with 2.5% or 5% DSS-containing water for 7 days and tap water for another 1 day (Fig. 7A). To confirm the effectiveness of modeling, we recorded body weights and clinical signs of the mice during the whole experiments. Compared with the control group, significant weight loss was observed in 2.5% and 5% DSS-treated groups (Fig. 7B). Mice treated with 5% DSS lost 8.6% of the body weight on the fourth day of modeling, and 1.2% of the body weight on the sixth day of modeling. 2.5% DSS-treated mice showed a milder trend of weight loss during the whole period, and lost 1.2% of the body weight on the sixth day of modeling, while the control group showed no significant body weight changes. On the other hand, mice defecation varied in different groups. 5% DSS-treated mice showed increased fecal viscosity on the fifth day of modeling, and there was blood in the stool on the very next day. Increased fecal consistency was observed in several 2.5% DSS-treated mice on the seventh day of modeling, while no defecation abnormality appeared in the control group. According to the clinical IBD scoring criteria, 5% DSS treatment induced severe colitis (3.4 points), 2.5% DSS treatment induced moderate colitis (1.67 points), and no colitis signs were found in the control group (Fig. 7C). These results suggested the successful modeling of severe and moderate colitis mice in our study from a clinical perspective.

### In vivo CT imaging of colitis mice

**Fig. 8.**
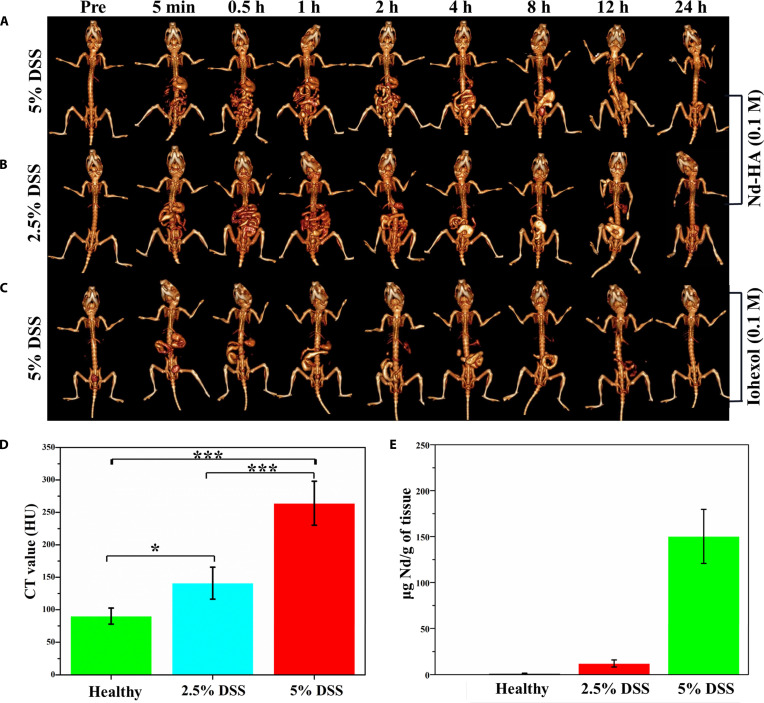
In vivo CT imaging of GI tract in colitis mice. Images of (A) 5% DSS and (B) 2.5% DSS-induced colitis mice after oral administration of 0.1 M Nd-HA NPs. (C) Images of 5% DSS-induced colitis mice after oral administration of 0.1 M iohexol. (D) CT values and (E) Nd content of healthy mice, moderate colitis mice (2.5% DSS), and severe colitis mice (5% DSS) 24 h post-Nd-HA NPs administration (without gastrointestinal contents). All values were expressed as mean ± SEM (*n* = 3), *P < 0.05, ***P < 0.001.

Subsequently, Nd-HA NPs were applied for CT imaging in 2.5% DSS-induced (moderate) and 5% DSS-induced (severe) colitis mice. Administration of iohexol to 5% DSS-treated mice was used as a comparison (*n* = 3). CT imaging was conducted at different time points (pre, 5 min, 0.5 h, 1 h, 2 h, 4 h, 8 h, 12 h, and 24 h). After Nd-HA NPs administration, no significant differences were found between healthy and colitis mice within the first 0.5 h, indicating that Nd-HA NPs were also able to depict the GI tract of colitis mice. However, the metabolism of Nd-HA NPs was much slower in colitis mice compared to healthy mice. Twelve hours after Nd-HA NPs treatment, there was a large amount of contrast agent residue in the lower GI tract of colitis mice, and the enrichment of Nd-HA NPs was observed in the colon of colitis mice 24 h post-administration (Fig. 8A and B and Fig. S10). Nd-HA NPs generated the strongest CT signals in 5% DSS-induced colitis mice 24 h post-administration.

As shown in Fig. S11, after oral administration of Nd-HA NPs, colon CT values of all the groups increased rapidly within 0 to 4 h and remained relatively stable during 4 to 12 h. At the 24-h time point, the CT values of the control group returned to baseline levels, whereas 2.5% and 5% DSS groups showed obviously higher CT values compared to the control groups, confirming that Nd-HA NPs displayed good targeting ability toward colitis. However, there was no contrast agent enrichment in iohexol and barium sulfate-treated colitis mice 24 h post-administration because they are not specific for inflammatory tissues (Fig. 8C and Fig. S12A). To verify whether the inflammation specificity of Nd-HA NPs derived from the CD44 targeting ability of HA unit, Nd-DTPA was administrated to 5% DSS-induced colitis mice. Negligible CT signals were observed in the colon region 24 h post-administration, suggesting that Nd-HA NPs achieved active targeting of inflammation by interacting with CD44 receptors via HA unit (Fig. S12b).

To further evaluate whether Nd-HA NPs had the potential to assess the severity levels of IBD, colon CT values of healthy mice and moderate and severe colitis mice were measured as the average of 3 consecutive slices, which were determined to be 90.19, 140.99 (*P* < 0.05, 95% CI = 3.8549 to 54.5984) and 264.07 (*P* < 0.001, 95% CI = 107.5962 to 138.5638), respectively (Fig. 8D). In addition, ICP-OES test of colon tissues showed that the Nd content in healthy mice, 2.5% DSS-treated mice, and 5% DSS-treated mice was 1.32, 12.07, and 150.30 μg Nd/g of tissue, respectively (Fig. 8E). Colon shortening is a common and clinically significant feature of IBD, primarily resulting from direct hyperosmotic damage to epithelial cells. Correlation analysis demonstrated a strong negative relationship between colon length and CT values (*R*^2^ = 0.994), supporting the use of CT imaging as a quantitative indicator of disease severity (Fig. S13). These results consistently proved that Nd-HA NPs effectively distinguished healthy mice, moderate colitis mice, and severe colitis mice, showing great potential in IBD diagnosis and grading.

**Fig. 9.**
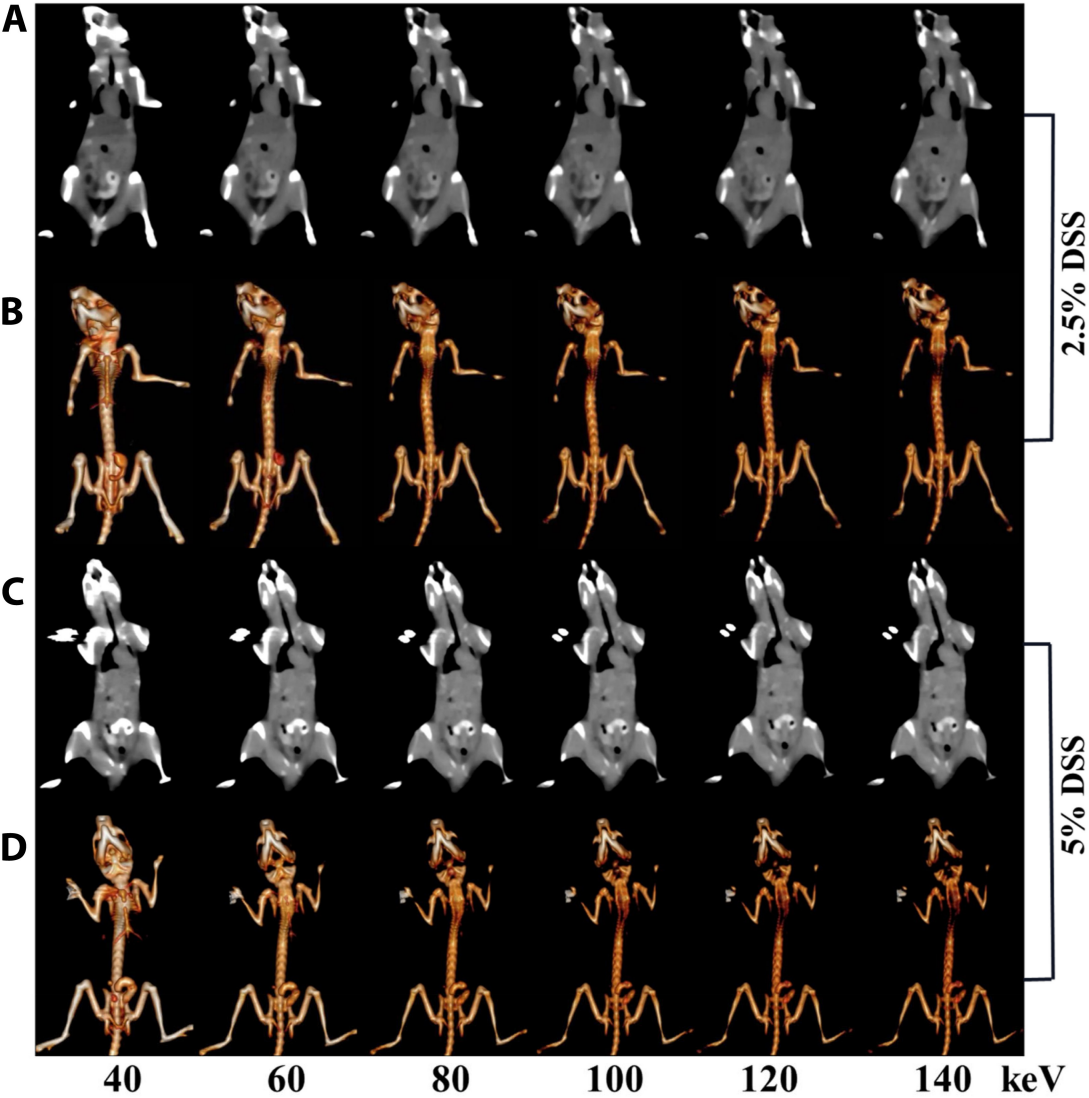
In vivo spectral CT imaging of GI tract in different colitis mice after oral administration of 0.1 M Nd-HA NPs for 24 h. (A) Gray-scale coronal CT images and (B) 3D reconstruction images of 2.5% DSS-induced colitis mice. (C) Gray-scale coronal CT images and (D) 3D reconstruction images of 5% DSS-induced colitis mice.

In order to achieve better imaging and grading of colitis, spectral CT imaging of Nd-HA NPs in 2.5% DSS-treated (moderate) and 5% DSS-treated (severe) colitis mice was performed 24 h post-administration. Virtual monochromatic energy images and corresponding gray-scale coronal images at 40, 60, 80, 100, 120, and 140 keV were obtained by post-processing the scanned CT images. Accumulation of Nd-HA NPs and strong spectral CT signals were best visualized at 40 keV in both groups. Image attenuation intensified progressively at higher monochromatic energies. CT values in the colons of moderate colitis mice attenuated sharply between 60 and 140 keV, and the outline of the GI tract became increasingly flurry during 80 to 140 keV (Fig. 9A and B and Figs. S14 and S15). In contrast, the GI structures of severe colitis mice remained visible at all monochromatic energies (Fig. 9C and D). These results showed that after Nd-HA NPs treatment, accumulation of the contrast agents was observed in the colon of both moderate and severe colitis mice at 24 h. More importantly, there were statistically significant differences in the CT values of the GI tract between moderate colitis mice and severe colitis mice. Therefore, we assumed that Nd-HA NPs could realize accurate diagnosis and grading of colitis in spectral CT imaging given their favorable inflammation targeting ability.

**Fig. 10.**
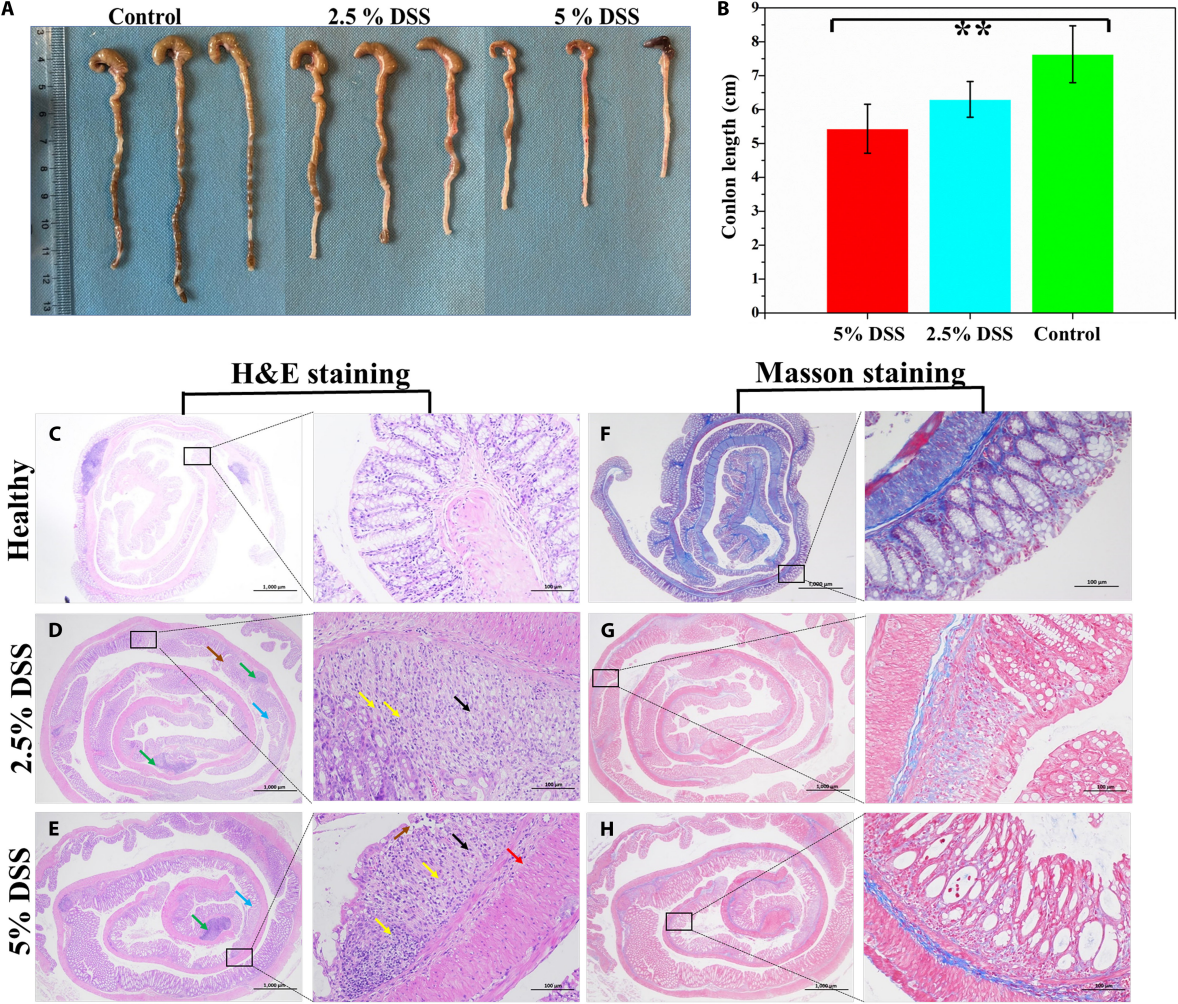
(A) Colon tissues isolated from healthy and colitis mice. (B) Colon lengths of different groups, values were expressed as mean ± SEM (*n* = 3), **P < 0.01. (C to E) H&E staining of healthy mice, 2.5% and 5% DSS-induced colitis mice. Brown arrows indicated epithelial cell detachment, yellow arrows indicated scattered lymphocytes and punctate granulocyte infiltration, blue arrows indicated intestinal glands dilatation, green arrows indicated lymphoid nodules, red arrows indicated infiltration of inflammatory cells to the submucosa, and black arrows indicated necrolysis and lysis of intestinal glands in the lamina propria, which were replaced by hyperplastic connective tissue. (F to H) Masson staining of healthy mice, 2.5% and 5% DSS-induced colitis mice. Scale bar: 200 μm.

To confirm the severity of colitis from a pathology aspect, healthy mice and 2.5% and 5% DSS-treated mice were dissected immediately after spectral CT imaging. Colon tissues were collected for length measurement since inflammation causes damage to the colon muscles and leads to shortening of colon length [40]. The average colon lengths of healthy mice, 2.5% DSS-treated mice, and 5% DSS-treated mice were 7.6, 6.3, and 5.4 cm (*P* < 0.01), respectively (Fig. 10A and B). As shown in Fig. 10C and F, no pathological damages were observed in healthy mice. In 2.5% DSS-induced colitis mice, there were small ulcers in the mucosal layer, accompanied by a small amount of epithelial cell detachment (brown arrow); more intestinal gland necrolysis and lysis in the lamina propria, which were replaced by hyperplastic connective tissue (black arrows); scattered lymphocytes with punctate granulocyte infiltration (yellow arrows); a few dilated intestinal glands (blue arrows); and a small number of lymphoid nodules (green arrows). The phenomenon verified that mice treated with 2.5% DSS had moderate colitis (Fig. 10D and G). In addition, 5% DSS-induced colitis mice showed multiple ulcers of the mucosal layer with epithelial cell detachment (brown arrow); more necrolyzed and lysed intestinal glands in the lamina propria, which were replaced by hyperplastic connective tissues (black arrow); scattered lymphocytes infiltrated with punctate granulocytes (yellow arrow); and a small number of inflammatory cells infiltrated the submucosal layer (red arrow), intestinal gland dilation (blue arrow), and lymphoid nodules (green arrow), confirming that 5% DSS treatment induced severe colitis (Fig. 10E and H). These results demonstrated the successful establishment of moderate colitis and severe colitis, which further validate the possibility of utilizing Nd-HA NPs to achieve colitis grading in spectral CT imaging.

### Pharmacokinetics

At 0.5 h after oral administration of Nd-HA NPs, a slight increase in Nd element content was observed in parenchymal organs of healthy mice. However, nearly complete metabolism occurred within 24 h, indicating that orally delivered Nd-HA NPs were efficiently cleared within 1 day without causing damage to vital organs (Fig. S16). Besides, Nd element levels in the stomach, small intestine, and large intestine were also measured, which increased markedly at 0.5 h post-administration and were almost metabolized within 24 h in the stomach, small intestine, and large intestine. These results collectively confirmed that Nd-HA NPs are largely cleared within 24 h post-administration and do not cause detectable harm in mice.

## Discussion

Given the difficulties in the early diagnosis and nonintrusive assessment of IBD, we developed the novel spectral CT contrast agent Nd-HA NPs. Firstly, Nd-HA NPs were prepared through a green one-pot reaction with the yield of 95%. Next, CCK-8 assay was performed on 4T1 and MCF-10A cells to test the cytotoxicity of Nd-HA NPs, and cell viability exceeded 80% at maximal Nd-HA NPs concentration (400 μg/ml) in both cells. Subsequently, we investigated the acute toxicity (1 day) and chronic toxicity (10 days) of Nd-HA NPs in mice. There were no significant differences between healthy mice and Nd-HA NPs-treated mice in regard to liver and kidney function indicators, demonstrating that 0.1 M Nd-HA NPs exerted no adverse effect on liver and kidney. In addition, pathological H&E staining revealed that Nd-HA NPs did not cause any damage to the vital tissues and organs, including heart, liver, spleen, lungs, kidneys, stomach, small intestine, and large intestine. These results indicated that Nd-HA NPs exhibited good biocompatibility and low toxicity, laying the foundation for further in vivo study.

Spectral CT imaging is widely used in abdominal diagnosis because it provides a series of monochromatic energy images, which allows for better visualization of anatomical structures, elimination of artifacts, and reduction of contrast agent dosage [41]. After oral administration of Nd-HA NPs, healthy mice, moderate colitis mice, and severe colitis mice were scanned and analyzed in both conventional and spectral CT imaging. In healthy mice, Nd-HA NPs demonstrated superior imaging capabilities compared to iohexol, including higher CT values, enhanced x-ray attenuation, and the ability to visualize the entire GI structures. In colitis mice, varying enrichment of Nd-HA NPs was detected in the colon of moderate and severe colitis mice 24 h post-administration, in stark contrast to minimal signal enhancement in iohexol-treated cohorts. This validated the superior contrast efficacy of Nd-HA NPs over clinical iohexol for both healthy and inflamed bowel visualization.

Additionally, there was a significant difference between the CT values of 2.5% and 5% DSS-treated mice (*P* < 0.001). It was worth mentioning that the difference of Nd-HA NPs enrichment in the colon of moderate and severe colitis was more pronounced in spectral CT imaging. For moderate colitis mice, spectral CT signals were visualized at 40 and 60 keV, and then faded gradually as the monochromatic energies increased. In contrast, in severe colitis mice, colon accumulation of Nd-HA NPs was clearly visible throughout the entire monochromatic energy levels of 40 to 140 keV. There is a correlation between the accumulation of lanthanide probes in lesions and the severity of the disease [42,43]. ICP-OES analysis of colon tissues also revealed that the content of Nd element was much higher in severe colitis mice compared to moderate colitis mice (*P* < 0.001). These results were consistent with the clinical symptoms and pathological IBD scores of the mice, solidly confirming the great potential of Nd-HA NPs in IBD early diagnosis and severity assessment.

However, there were some shortcomings in our experiments. We chose female BALB/c mice (6 to 8 weeks old) instead of rabbits or dogs. In this way, the simulated colitis models and the biological results may not reflect the real situation of human diseases. We employed DSS-induced BALB/c mice to establish a colitis model. This approach is widely adopted for its high reproducibility and relevance to inflammation studies. However, the pathogenesis of colitis is complex, and drug-induced colitis does not fully replicate human disease. In addition, we have only completed the toxicity evaluation of acute and subacute toxicity for Nd-HA NPs; long-term and repeated toxicity investigation is essential for comprehensive preclinical safety assessment. As a result, we will further investigate the toxicity and imaging ability of Nd-HA NPs in other animal models. In conclusion, Nd-HA NPs had good structural stability, low toxicity, and excellent imaging ability, meeting the requirement of spectral CT imaging with much lower dosage compared to iohexol. Most importantly, Nd-HA NPs had the potential to achieve specific imaging of colitis and determine the severity of inflammation by observing the accumulation of Nd-HA NPs among healthy, moderate colitis, and severe colitis mice in spectral CT imaging.

In summary, we have synthesized the Nd-HA NPs via a facile and green one-pot reaction. Nd-HA NPs had the advantages of easy synthesis, good stability, excellent biocompatibility, and superior x-ray absorption and attenuation compared to the clinical contrast agent iohexol. In addition, Nd-HA NPs not only clearly depicted the complete GI structures but also effectively distinguished healthy mice, moderate colitis mice, and severe colitis mice especially in spectral CT imaging, showing the ability to accuracy diagnose and evaluate the severity of colitis. Therefore, such nanoparticles represent a novel option for the future development of spectral CT contrast agents to advance the early clinical diagnosis and grading of IBD.

## Data Availability

All data can be provided as needed.
