# Supplementary material for: Spectral Computed Tomography Diagnosis of Inflammatory Bowel Disease with Neodymium-Hyaluronic Acid Nanoparticles
Source: Biomater Res. 2026 Mar 25;30:0301. doi: 10.34133/bmr.0301 (PMC13014110; doi:10.34133/bmr.0301)
Supplement: Supplementary 1 — Figs. S1 to S16 Tables S1 and S2 [file bmr.0301.f1.docx]

Supplementary information for

**Spectral computed tomography diagnosis of inflammatory bowel disease with Neodymium-hyaluronic acid nanoparticles**

Xin Zhu^1, 3†^, Wenqian Ru^1†^, Lin Luo^1†^, Liu Zhao^1^, Didi Gu^1^, Xi Deng^1^, Yihan Wang^1^, Pengyi Huang^1^, Qiuyu Meng^2^*, Chunmei Yang^1, 3^* and Lu Yang^1, 3^*

*^1^ Department of Radiology, The Affiliated Hospital, Southwest Medical University, NO.25, Taiping Road, Jiangyang District, Luzhou, 646000, Sichuan, China*

*^2^ Key Laboratory of Pollution Exposure and Health Intervention of Zhejiang Province, Interdisciplinary Research Academy (IRA), Zhejiang Shuren University, Hangzhou 310015, China*

*^3^ Precision Imaging and Intelligent Analysis Key Laboratory of Luzhou, Southwest Medical University, Luzhou, 646000, Sichuan, China*

*^†^These three authors contributed equally to this work*

**Correspondence to: Qiuyu Meng,* [*qiuyumeng@zjsru.edu.cn*](mailto:qiuyumeng@zjsru.edu.cn)*; Chunmei Yang, ycm020@swmu.edu.cn; Lu Yang,* [*yanglu@swmu.edu.cn*](mailto:yanglu@swmu.edu.cn)


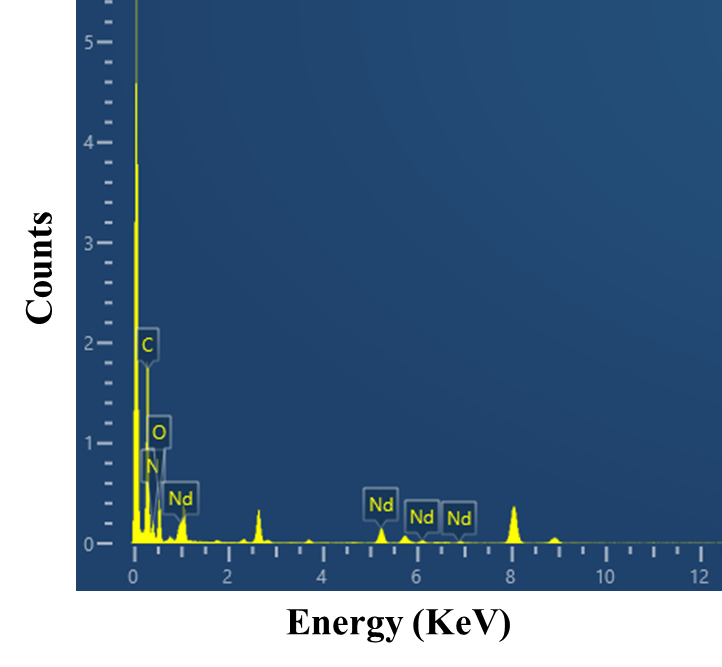


**Fig. S1** EDS spectrum of Nd-HA NPs.


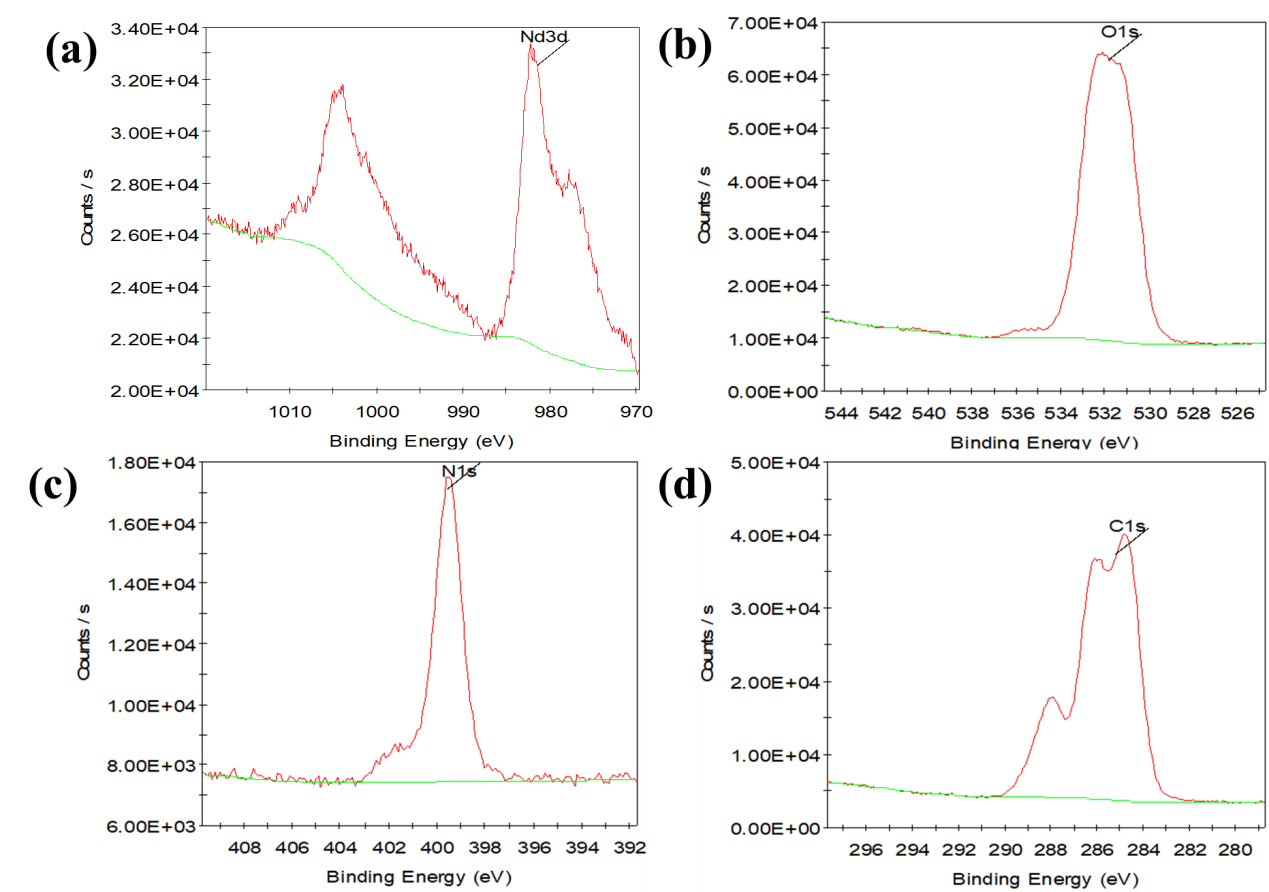
**Fig. S2** High-resolution XPS spectra of (a) Nd 3d; (b) O 1s; (c) N 1s; (d) C 1s of Nd-HA NPs.


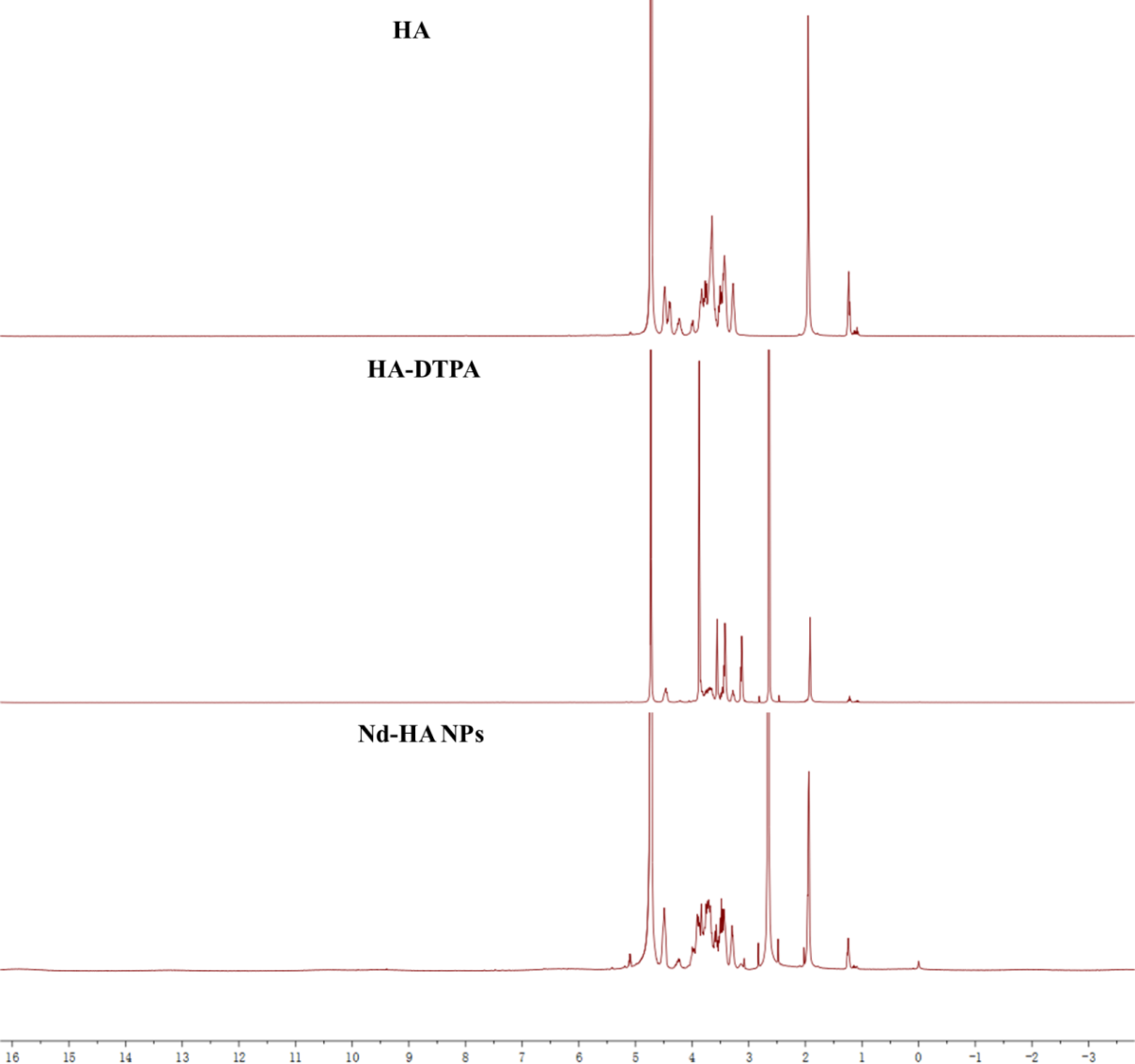


**Fig. S3** ^1^H-NMR spectra of HA, HA-DTPA, and Nd-HA NPs.


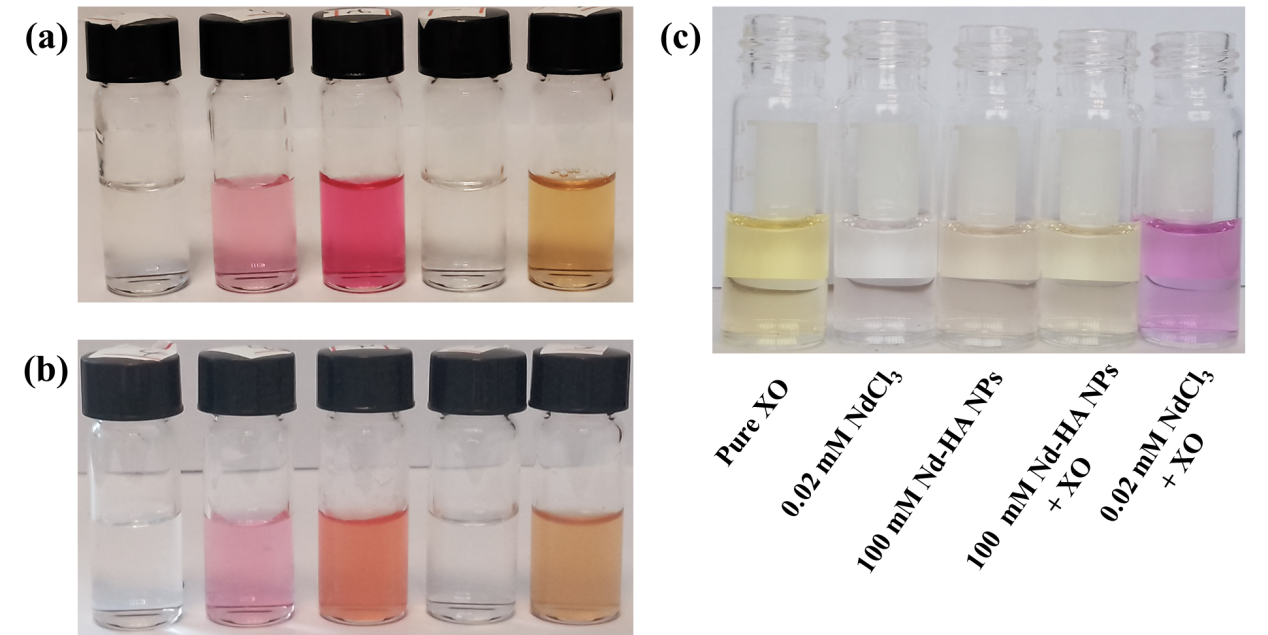


**Fig. S4** Photographs of Nd-HA NPs solutions in different media (1 mg/mL, from left to right: normal saline, RPMI-1640, DMEM, PBS and FBS, 37°C) taken at (a) 1^st^ and (b) 7^th^ days; (c) Nd^3+^ leakage measurement of Nd-HA NPs and NdCl_3_ in PBS by using the XO indicator. The solutions from left to right were pure XO solution (0.2 %), 0.02 mM NdCl_3_ solution, 100 mM Nd-HA NPs solution, 100 mM Nd-HA NPs solution containing the XO indicator, and 0.02 mM NdCl_3_ solution containing the XO indicator.


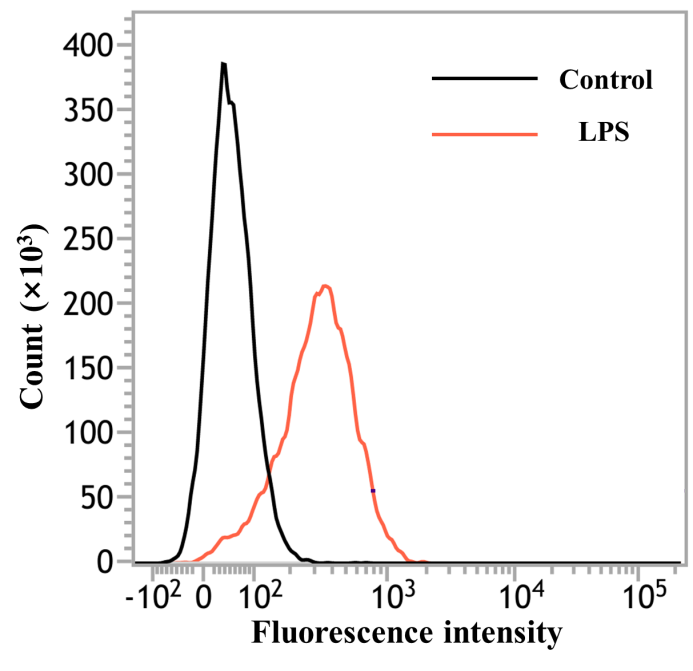


**Fig. S5** Flow cytometric analysis of CD44 expression in LPS-treated RAW264.7 cells.

**
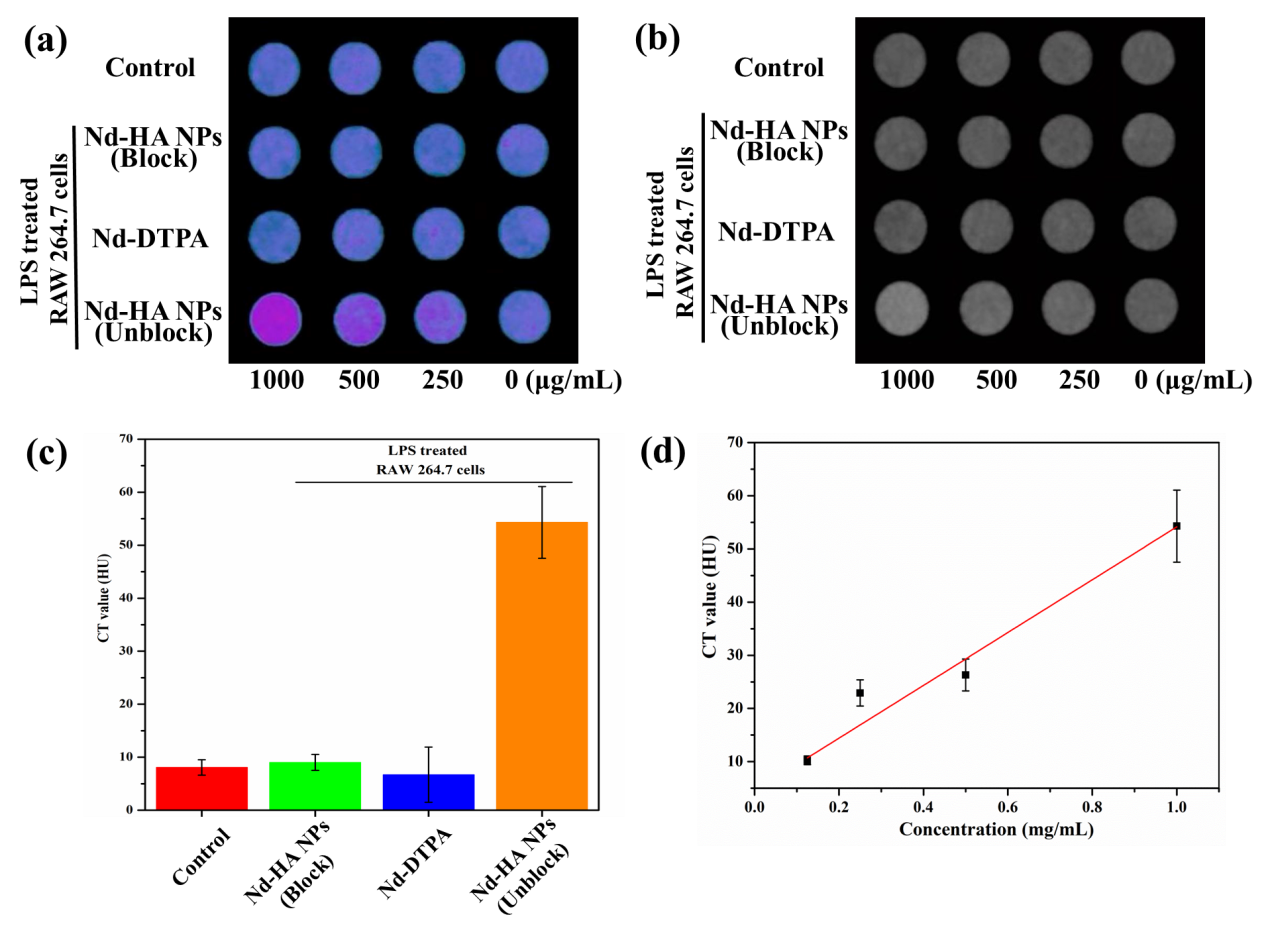
**

**Fig. S6** (a) Pseudo-color and (b) grayscale images of cellular-targeted CT scans; (c) CT values of RAW264.7 cells under different treatments; (d) Correlation analysis between the concentration of Nd-HA NPs and the CT values of LPS-treated RAW264.7 cells.


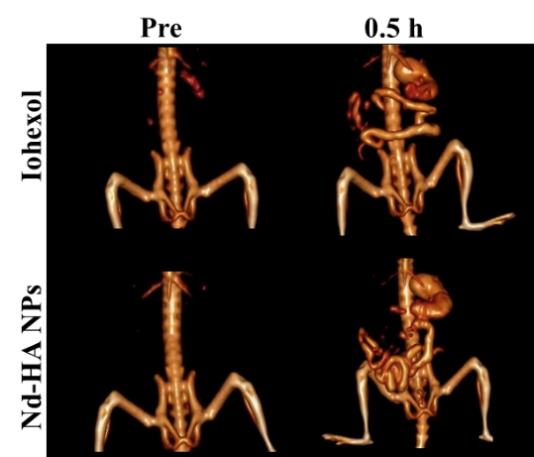


**Fig. S7** Magnified CT images of normal mice pre and 0.5 h post oral administration of iohexol and Nd-HA NPs.


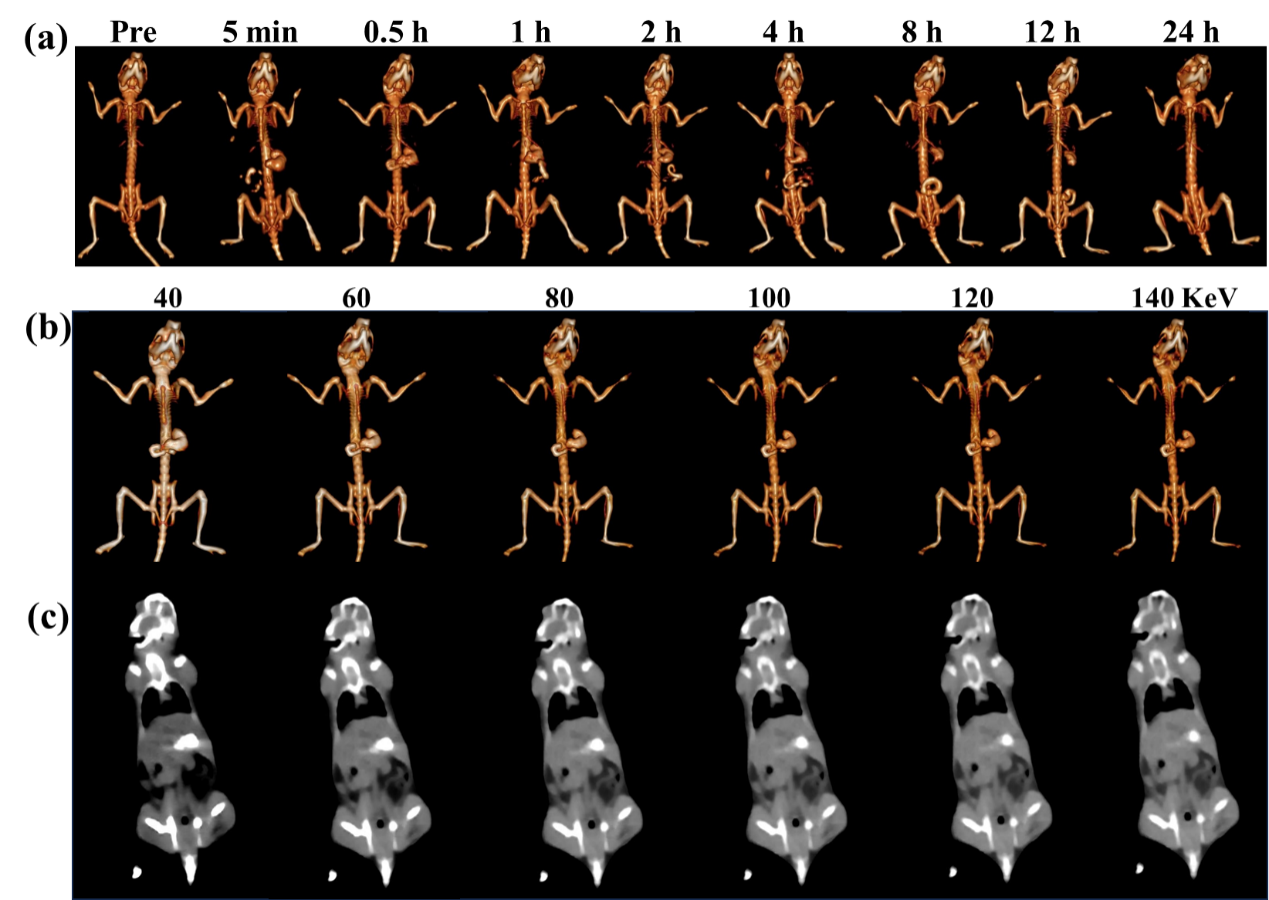


**Fig. S8** (a) CT images of healthy mice before and after barium sulfate administration; (b) 3D reconstruction images and (c) Gray-scale coronal CT images of healthy mice after barium sulfate administration for 0.5 h conducted under different monochromatic energies.


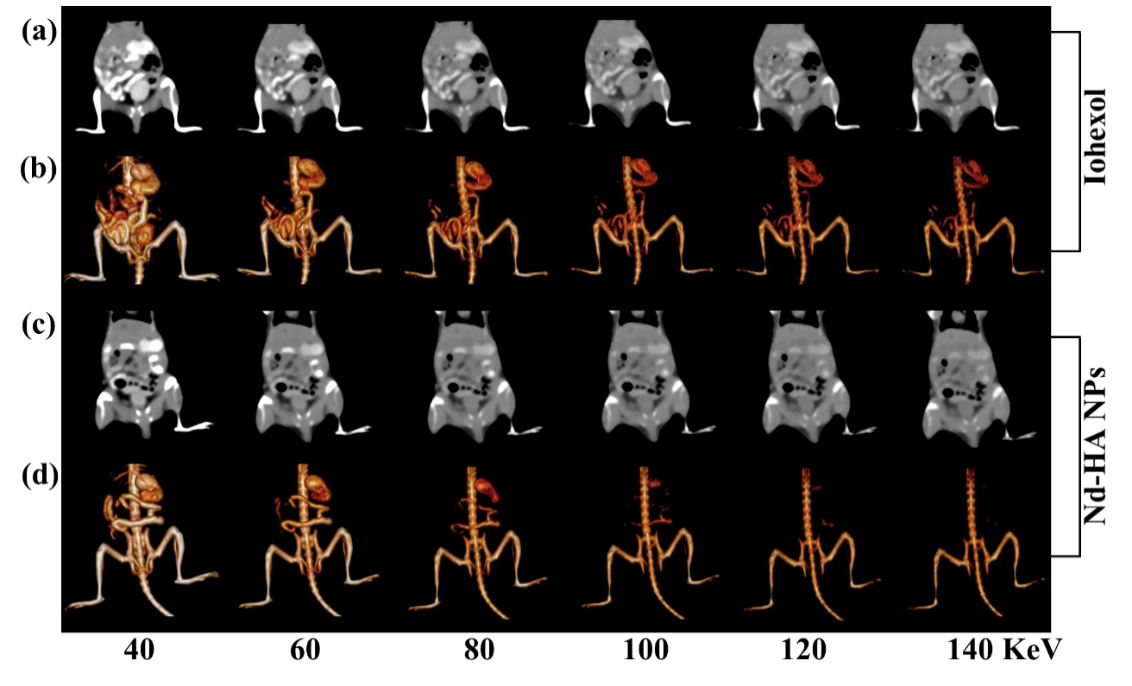


**Fig. S9** Magnified CT images of healthy mice at 0.5 h post oral administration of (a, b) Nd-HA NPs and (c, d) iohexol Nd-HA NPs.


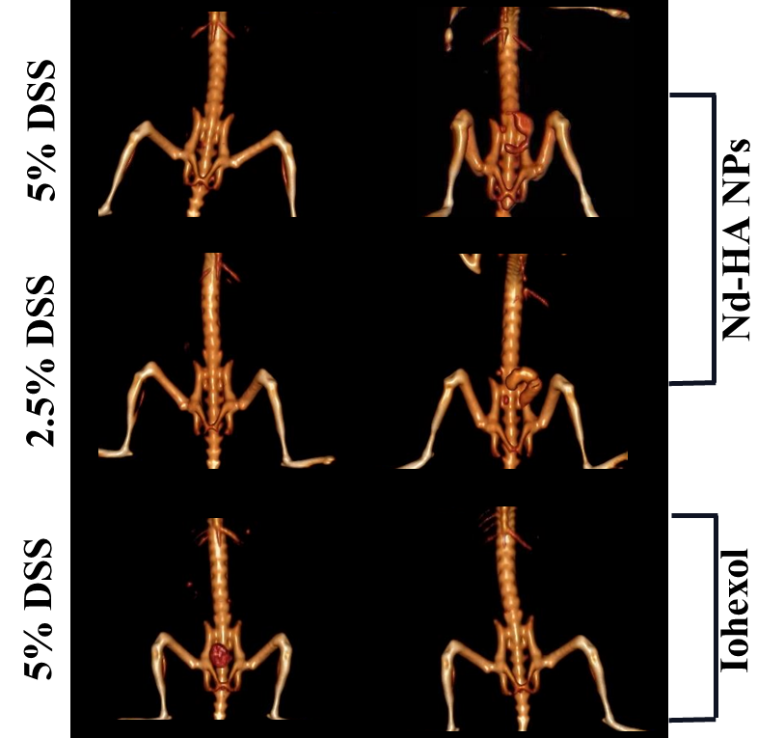


**Fig. S10** Magnified CT images of 5% DSS-induced colitis mice pre and 24 h after oral administration of Nd-HA NPs and iohexol, and 2.5% DSS-induced colitis mice at 24 h after oral administration of Nd-HA NPs.

**

**

**Fig. S11** Colon CT values of healthy mice, 2.5% and 5% DSS-treated mice after administrating with Nd-HA NPs within 24 h.


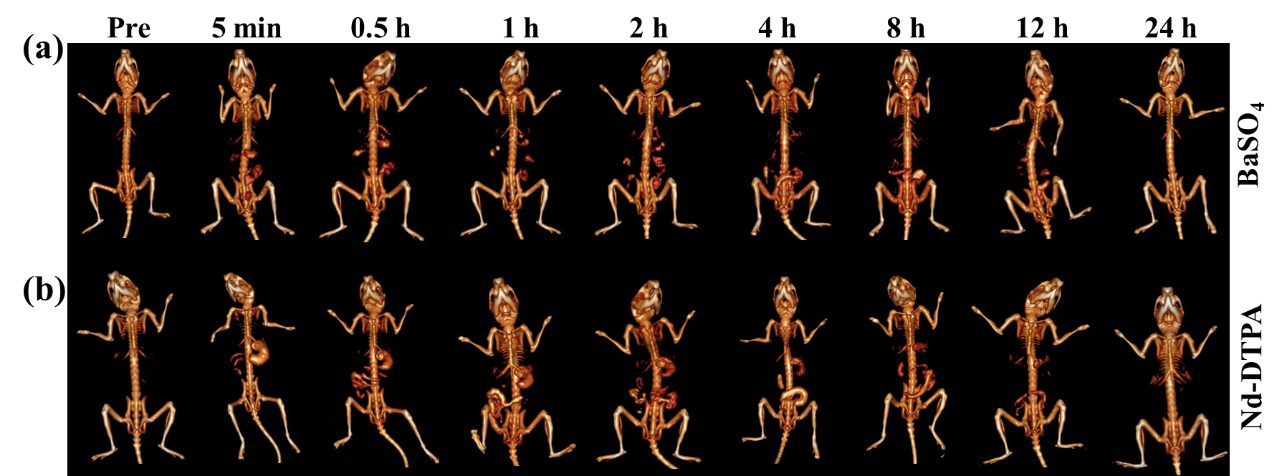


**Fig. S12** *In vivo* CT imaging of GI tract in colitis mice. (a) Images of 5% DSS-induced colitis mice after oral administration of 0.1 M barium sulfate; (b) Images of 5% DSS-induced colitis mice after oral administration of 0.1 M Nd-DTPA.


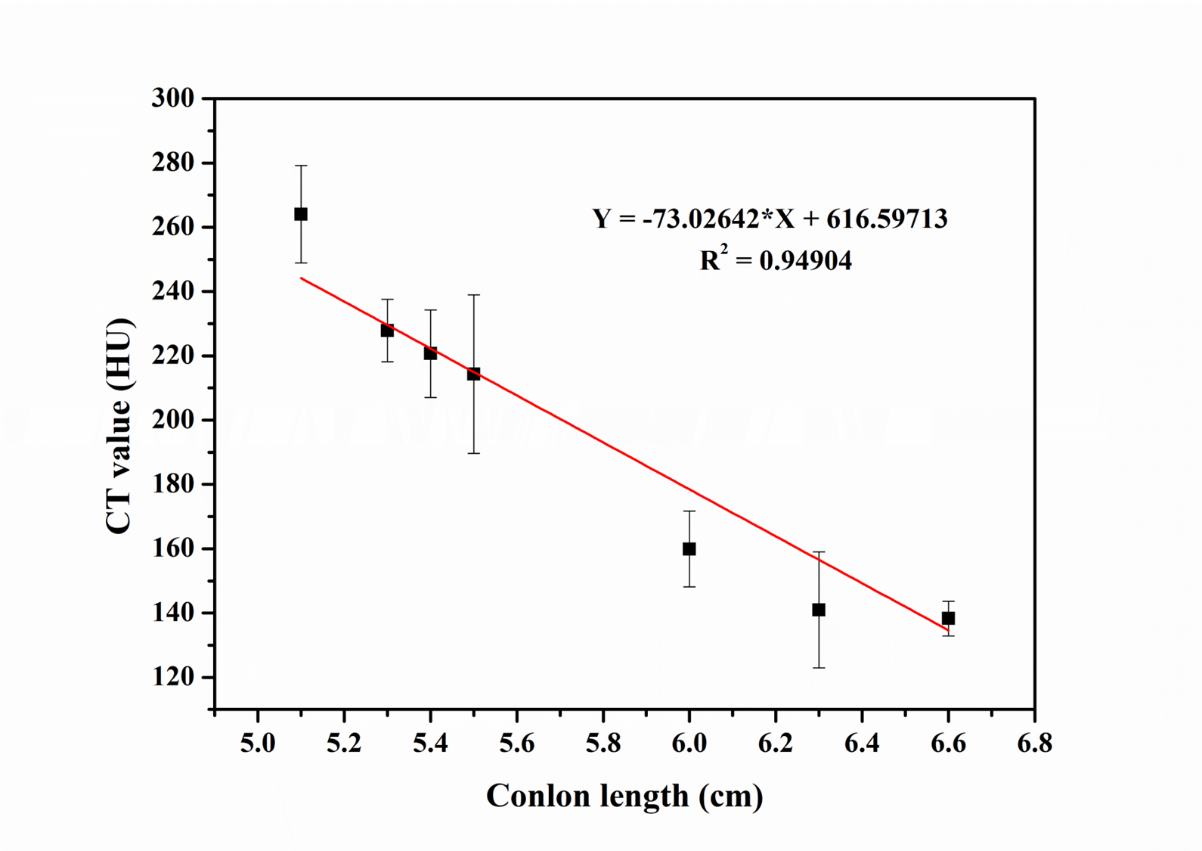


**Fig. S13** Correlation analysis between colon length and colon CT values.


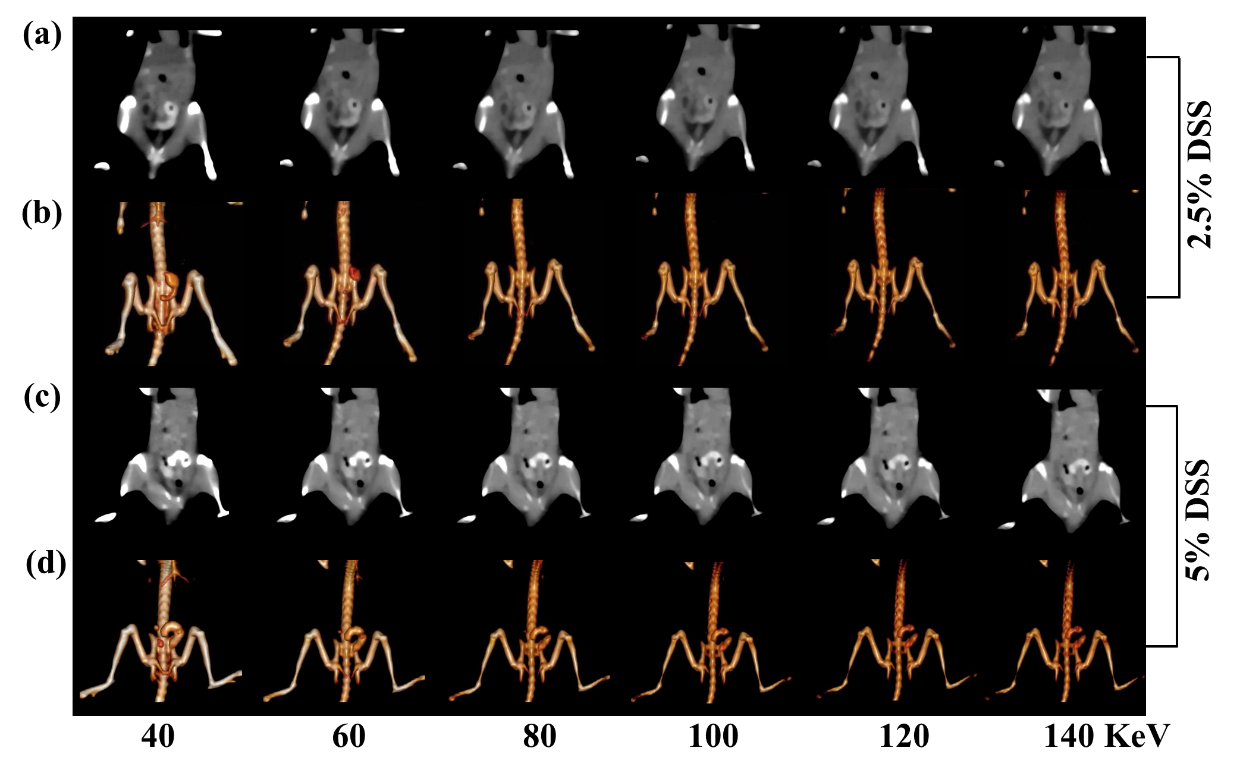


**Fig. S14** Magnified CT images of colitis mice induced by (a-b) 2.5% and (c-d) 5% DSS at 24 h post oral administration of Nd-HA NPs.





**Fig. S15** Colon CT values of healthy mice, 2.5% and 5% DSS-treated mice after administrating with Nd-HA NPs under different monochromatic energies.





**Fig. S16** Nd element content in heart, liver, spleen, lung, kidney, stomach, small intestine, and large intestine pre, 0.5 h and 24 h post oral administration of Nd-HA nanoparticles.

**Table S1**. Leakage rate of Nd^3+^ in simulated body fluid.

|  | **pH=2.2** | **pH=8.0** |
| --- | --- | --- |
| Nd-HA NPs | 0.15 % | 0.08 % |

**Table S2.** X-ray mass attenuation coefficient at 100 keV (cm^2^g^-1^) of different elements.

| element | Atomic number (Z) | K-edge energy (keV) | X-ray mass attenuation coefficient at 100 keV (cm^2^g^-1^) |
| --- | --- | --- | --- |
| I | 53 | 33.2 | 1.94 |
| Ba | 56 | 37.4 | 2.20 |
| La | 57 | 38.9 | 2.32 |
| Ce | 58 | 40.4 | 2.44 |
| Pr | 59 | 42.0 | 2.59 |
| Nd | 60 | 43.6 | 2.69 |
| Pm | 61 | 45.2 | 2.84 |
| Sm | 62 | 46.8 | 2.90 |
